# Genomic Characterization of a South American *Phytophthora* Hybrid Mandates Reassessment of the Geographic Origins of *Phytophthora infestans*

**DOI:** 10.1093/molbev/msv241

**Published:** 2015-11-17

**Authors:** Michael D. Martin, Filipe G. Vieira, Simon Y.W. Ho, Nathan Wales, Mikkel Schubert, Andaine Seguin-Orlando, Jean B. Ristaino, M. Thomas P. Gilbert

**Affiliations:** ^1^Centre for GeoGenetics, Natural History Museum of Denmark, University of Copenhagen, Copenhagen, Denmark; ^2^Department of Integrative Biology, Center for Theoretical Evolutionary Genomics, University of California, Berkeley; ^3^School of Biological Sciences, University of Sydney, Sydney, NSW, Australia; ^4^Department of Plant Pathology, North Carolina State University; ^5^Trace and Environmental DNA Laboratory, Department of Environment and Agriculture, Curtin University, Perth, WA, Australia

**Keywords:** *Phytophthora infestans*, *Phytophthora andina*, plant disease, population genomics, phylogenomics, pathogen

## Abstract

As the oomycete pathogen causing potato late blight disease, *Phytophthora infestans* triggered the famous 19th-century Irish potato famine and remains the leading cause of global commercial potato crop destruction. But the geographic origin of the genotype that caused this devastating initial outbreak remains disputed, as does the New World center of origin of the species itself. Both Mexico and South America have been proposed, generating considerable controversy. Here, we readdress the pathogen’s origins using a genomic data set encompassing 71 globally sourced modern and historical samples of *P. infestans* and the hybrid species *P. andina*, a close relative known only from the Andean highlands. Previous studies have suggested that the nuclear DNA lineage behind the initial outbreaks in Europe in 1845 is now extinct. Analysis of *P. andina*’s phased haplotypes recovered eight haploid genome sequences, four of which represent a previously unknown basal lineage of *P. infestans* closely related to the famine-era lineage. Our analyses further reveal that clonal lineages of both *P. andina* and historical *P. infestans* diverged earlier than modern Mexican lineages, casting doubt on recent claims of a Mexican center of origin. Finally, we use haplotype phasing to demonstrate that basal branches of the clade comprising Mexican samples are occupied by clonal isolates collected from wild *Solanum* hosts, suggesting that modern Mexican *P. infestans* diversified on *Solanum tuberosum* after a host jump from a wild species and that the origins of *P. infestans* are more complex than was previously thought.

## Introduction

Species in the oomycete genus *Phytophthora* (plant destroyer) are important pathogens on many plants in agricultural, forest and aquatic environs, and new species are being described at an accelerating pace (Hansen et al. 2012; Guha Roy and Grünwald 2015). *Phytophthora* spp. have large biological and agronomic impacts and are pathogenic on natural populations of many host species (e.g., *P. ramorum*; Rizzo et al. 2005), as well as important crops such as tomatoes, potatoes, soybeans, alfalfa, peppers and cocoa (Erwin and Ribeiro 1996). Although the major locales of diversification of their primary hosts often have been identified, centers of origin of many *Phytophthora* species are unknown (Hansen et al. 2012).

Among *Phytophthora* species*, P. infestans* is perhaps the most widely known. Its reputation arises both from its destructive potential as a plant pathogen that was once responsible for great human loss, and to a particular ability to inspire scientific controversy, for example, in its early identification as the causal agent of a plant disease, as well as its highly disputed New World origin (Bourke 1991). Placed within *Phytophthora* clade 1c, the species likely originated in the New World tropics, the center of diversity for many host species (Douglas and Manos 2007; Olmstead et al. 2008; Serrano-Serrano et al. 2010). Specifically where this center of origin lies, however, remains debated (Abad ZG and Abad JA 1997; Kroon 2010), with different lines of evidence pointing to either central Mexico (Goss et al. 2014) or the South American Andes (Gómez-Alpizar et al. 2007). Important for the debate is that *P. infestans* has the capacity for both sexual and asexual reproduction. Sexual populations are sources of novel *P. infestans* diversity and were first discovered in Mexico (Drenth et al. 1993; Goodwin 1997), although asexual lineages are also highly adaptable due to an unstable, repeat-rich genome (Haas et al. 2009) and possibly frequent mitotic recombination (Goodwin 1997; Chamnanpunt et al. 2001; Balloux et al. 2003).

At least four lines of evidence have been interpreted as supporting a Mexican origin. First, genetically diverse, sexually reproducing populations have been observed in the Toluca Valley in the highlands of central Mexico. Second, resistance genes against *P. infestans* exist in wild Mexican potato species such as *Solanum demissum*. Third, the closest relatives of *P. infestans* yet identified (*P. ipomoeae* and *P. mirabilis*) are endemic in Mexico (Grünwald and Flier 2005). Finally, although both mating types can be found in various locales within Andean South America, the mating types are geographically isolated, and no sexual populations have been observed there (Perez et al. 2001; Adler et al. 2002; Forbes et al. 2013). Goss et al. (2014) recently performed a Bayesian phylogeographic analysis of four nuclear markers and concluded a Mexican origin for the species and the entire clade 1c. Although measures of nucleotide diversity were higher in Andean populations at all but one locus, the higher diversity was ascribed to hybridization or admixture in South America.

However, compelling evidence also points to an origin in the South American Andes, which is a center of origin of cultivated potatoes and where historical observations of endemic late blight in Andean Solanaceae hosts were reported (Berkeley 1846; De Bary 1863; Bourke 1964; Abad ZG and Abad JA 1997). Second, phylogenetic analyses of mitochondrial genome sequences from *P. infestans* have identified two major haplogroups, one of which (type II) exists mostly within South American isolates (Avila-Adame et al. 2006; Martin et al. 2014). Third, Gómez-Alpizar et al. (2007) used coalescent analyses to argue that the oldest mutations in analyzed nuclear and mitochondrial loci were present only in South American isolates, and that initial migration out of South America was the most likely scenario. Fourth, elevated allelic diversity of the US-1 clonal lineage in Ecuador has been suggested as evidence that this lineage is endemic there (Oliva et al. 2007; Forbes et al. 2013).

A second debated question relates to the source of the historical genotypes that first destroyed potato crops on a global scale. Although Mexico is the clear source of recent migrations of more virulent genotypes into Europe and the United States (Fry 2008), the ancestral geographic source of the first *P. infestans* introductions into the United States/Europe in the 1840s is still unresolved (Bourke 1964; Abad ZG and Abad JA 1997; Grünwald and Flier 2005; Birch and Cooke 2013). Analysis of ancient DNA (aDNA) has been useful for resolving questions about the migratory history of *P. infestans* by providing a direct window into past events. For example, an introduction of *P. infestans* carrying the US-1/Ib mitochondrial lineage was suspected to have caused the famine-era outbreaks in Europe, until polymerase chain reaction (PCR) and genomic sequencing of historical *P. infestans* samples embedded in herbarium-sourced potato leaves revealed that it was an entirely different lineage (Ristaino et al. 2001; May and Ristaino 2004). More recent aDNA studies reported genomic data from additional historical samples of *P. infestans* from western and northern Europe (Martin et al. 2013; Yoshida et al. 2013; Martin et al. 2014). Assembly and phylogenetic analysis of whole mitogenomes from historical *P. infestans* assigned these 19th-century samples to a novel mitogenomic lineage called HERB-1, which was originally proposed as extinct (Birch and Cooke 2013; Yoshida et al. 2013), but then shown to be extant in *P. infestans* isolates from both Mexico and Ecuador (Martin et al. 2014).

Ongoing failure in resolving these questions using multilocus genotyping and conventional phylogeographic analyses mandates the adoption of new approaches. The key to the origins of *P. infestans* may lie in *P. andina* (Gómez-Alpizar et al. 2008; Oliva et al. 2010), a close Andean relative hypothesized to have arisen through multiple hybridizations between *P. infestans* and an as yet undescribed species within *Phytophthora* clade 1c (Goss et al. 2011; Blair et al. 2012). Identified to date only within a limited geographic range in the highlands of Ecuador and Peru (Adler et al. 2004), *P. andina* is a pathogen on various *Solanum* species, and in some South American locales occurs sympatrically with *P. infestans* on *S. betaceum* and *S. muricatum* (Adler et al. 2004; Gómez-Alpizar et al. 2008; Pérez 2009; Oliva et al. 2010; Cárdenas et al. 2011; Forbes et al. 2013). A close phylogenetic relationship between *P. andina* and *P. infestans* has also been used as evidence for an Andean origin of both species (Adler et al. 2004; Gómez-Alpizar et al. 2007). Although some debate its status as a novel species (Cárdenas et al. 2011; Forbes et al. 2011; Oliva et al. 2010), interspecies hybridization is common in *Phytophthora* and is presumed to have been a major driver of evolution within the genus. This is because hybridization events can lead to changes in host range (host jumping), the loss of sex, and subsequent speciation (Goodwin and Fry 1994; Brasier 2000; Grünwald and Flier 2005; Raffaele et al. 2010; Goss et al. 2011; Blair et al. 2012).

Of key relevance to the origin of *P. infestans* is the observation that *P. andina* isolates contain two highly divergent mtDNA haplotypes (Adler et al. 2004). Although one (Ic) is clearly distinct from *P. infestans* lineages, the other (Ia) is closely related to the historical European haplotypes that were recently assigned to the HERB-1 mitogenome lineage (Yoshida et al. 2013; Lassiter et al. 2015). We hypothesized that the underlying reason for this may be relevant for a full understanding of *P. infestans* origins, so we assembled and analyzed a genomic data set of globally sourced *P. infestans* and *P. andina* samples. To resolve the aforementioned open questions in *P. infestans* population biology and to characterize its New World populations as potential sources of the historical European introductions, here we present phylogenetic and population genetic analyses of 57 nuclear genomes and 68 mitochondrial genomes from clade 1c *Phytophthora* species.

## Results

### Phylogenomic Analysis of Mitogenome Sequences

**Fig. 1.**
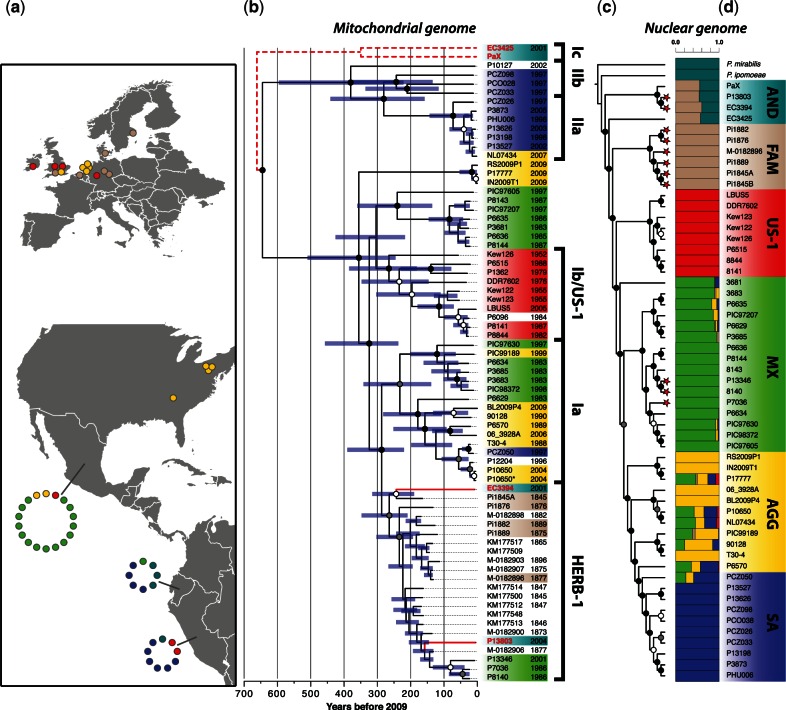
Relationships within a global sampling of historical and present-day *Phytophthora infestans* genomes. (*a*) Map of sample collections for which nuclear genomic data were analyzed. Circle colors indicate the samples’ nuclear genetic cluster assignment in panel (d). (*b*) Chronogram from Bayesian phylogenetic analysis of *P. infestans* and *P. andina* mitogenomes. Mitogenome sequences were calibrated by tip (collection) dates. Red branches and labels indicate *P. andina* isolates. The dashed lines illustrate the phylogenetic position of two type Ic *P. andina* mitogenomes that were not included in this phylogenetic reconstruction because they did not cluster with the *P. infestans* sequences and so violate the coalescent assumptions of the BEAST analysis. Asterices indicate sequence reads from Yoshida et al. (2013). Node support indicated by filled circles: Black, 90–100% support; gray, 75–89% support; white, 50–74% support. Blue bars indicate 95% highest posterior density intervals of node-age estimates (not shown for root node). Colored boxes indicate genetic cluster from panel (*d*). (*c*) Nuclear genome ML phylogeny. Node support was estimated from 100 bootstrap replicates. Tips with red stars indicate samples belonging to the HERB-1 mitogenomic lineage. (*d*) Admixture bar plot showing cluster assignments for *K* = 6 ancestral populations. The labels assigned to each genetic cluster (AND, FAM, US-1, MX, AGG, SA) are defined in the Results section of the main text.

We generated genomic data from 31 modern and historical samples and assembled sequence data from all previously published *P. infestans* genomic data sets, creating the largest globally distributed sample of *P. infestans* genomic data yet amassed (fig. 1*a* and supplementary table S1, Supplementary Material online). We then subjected putative mitogenomic reads to iterative, reference-guided assembly, producing mitogenome assemblies with mean read depths of over 200× (supplementary table S2, Supplementary Material online). The mitogenome phylogeny is consistent with previous estimates based on smaller data sets, displaying the well-known type I/type II divergence that segregates most South American isolates (fig. 1*b*).

Mitogenomes of *P. andina* isolates EC3425 and PaX diverged early from the *P. infestans* clade, supporting previous observations that these lineages had their origins in hybridization events in which the mtDNA donor was an unknown clade 1c *Phytophthora* spp. (Lassiter et al. 2015). Mitogenomes from 19th-century European samples of *P. infestans* have previously been assigned to a distinct lineage called HERB-1, which persists in extant isolates of *P. infestans* from Mexico and Ecuador (Martin et al. 2014). Remarkably, the lineage also persists in Ecuadorian isolates of *P. andina* EC3394 and P13803, both of which originate from *S. betaceum* hosts. A single A to G polymorphism is private to, and diagnostic of, all HERB-1 lineage samples (supplementary fig. S1, Supplementary Material online). Nodes uniting HERB-1 isolates of *P. andina* with their closest relatives are substantially younger than the most recent common ancestor (MRCA) of all HERB-1 sequences (264 years, 95% highest posterior density interval [HPD]: 220–390 years) and are assigned relatively young ages of 243 years (95% HPD interval: 190–315) for EC3394 and 158 years (95% HPD interval: 132–193) for P13803 (fig. 1*b*).

### Phylogenomic Analysis of Nuclear Genome Sequences

Nuclear genomes were sequenced to between 0.2× and 30.6× mean read depth over the entire approximately 240-Mb *P. infestans* T30-4 assembly (supplementary fig. S2, Supplementary Material online). Whole-genome phylogenetic analysis identifies six major lineages encompassing the *P. infestans* and *P. andina* samples (fig. 1*c* and supplementary fig. S3, Supplementary Material online) that we term: *P. andina* isolates (AND); famine-era European historical samples all carrying the HERB-1 mitochondrial lineage (FAM); US-1 clonal lineage samples, including three collected from the United Kingdom in the 1950s, all of which carry the Ib mitochondrial lineage (US-1); isolates largely collected from Mexico (MX); isolates largely collected from South America (SA); and various recently introduced, aggressive isolates of global origin (AGG), including recent US lineages.

Consistent with previous work (see Fry et al. 2015), some of the recently introduced aggressive strains in both Europe (06_3928A) and the United States appear to have a Mexican origin, as they share an MRCA with the largely Mexican MX clade, and Mexican isolates PIC99189 and P10650 segregate with this group (fig. 1*c*). For the same reasons, a Mexican origin for the SA isolates is also likely (Goss et al. 2014). Notably, the FAM and US-1 lineages fall in a highly supported position at the base of *P. infestans*, and US-1 is not derived from the MX clade. Two other phylogenomic estimation methods produced consistent topologies (supplementary figs. S4 and S5, Supplementary Material online). Although not specifically mentioned in their text, the results of Yoshida et al. (2013) showed that interclade relatedness of the mitogenome is inconsistent with that of the nuclear genome. We confirm these observations, especially for samples carrying the HERB-1 mitogenome lineage (fig. 1*b*).

We also produced a multilocus age estimate for nodes of our nuclear phylogeny for comparison with estimates from mitogenomic data presented by Martin et al. (2014) and Yoshida et al. (2013). We examined the mean age of the node uniting all FAM-cluster specimens as well as the root node uniting all *P. infestans* (including ingroup *P. andina* isolates when appropriate) at all nuclear gene alignments for which the analysis produced a “precise” estimate. Based only on the 155 genes with precise estimates, the mean of the median estimates for these ages yields a date of the MRCA of 1558 CE (Common Era) for the root node and 1590 CE for the famine-era outbreak clade. This supports the observation of Yoshida et al. (2013) that the estimated age of the *P. infestans* root node corresponds well with the earliest European activity in South America, and further suggests that the genotypes first introduced to 19th-century Europe originated shortly after the species itself diversified.

### Admixture Proportion Estimates

A history of movement between the disparate global locales considered in our samples is problematic for population genetic inference. Thus, we used genotype likelihoods to estimate population structure, assigning individual nuclear genomes to ad hoc genetic clusters without considering the actual geographic origins of collections. The largest peak of Δln(*K*) occurred at *K* = 2 (supplementary fig. S6, Supplementary Material online), for which all 19th-century European and US-1/Ib clonal lineage samples form a well-defined cluster with isolates of *P. andina*, which share 30–40% ancestry with an outgroup cluster containing only *P. ipomoeae* and *P. mirabilis* (supplementary fig. S7, Supplementary Material online). At greater values of *K*, the *P. andina* samples continue to show a mixed ancestry that includes both the outgroup species and the famine-era specimens, which provides further evidence that *P. andina* is a hybrid of an FAM genotype and an outgroup species. When *K* = 6, the genetic clusters generally agree with the topology of the phylogenetic tree (fig. 1*c* and *d*).

### Clonal Reproduction and Genomic Heterozygosity

**Fig. 2.**
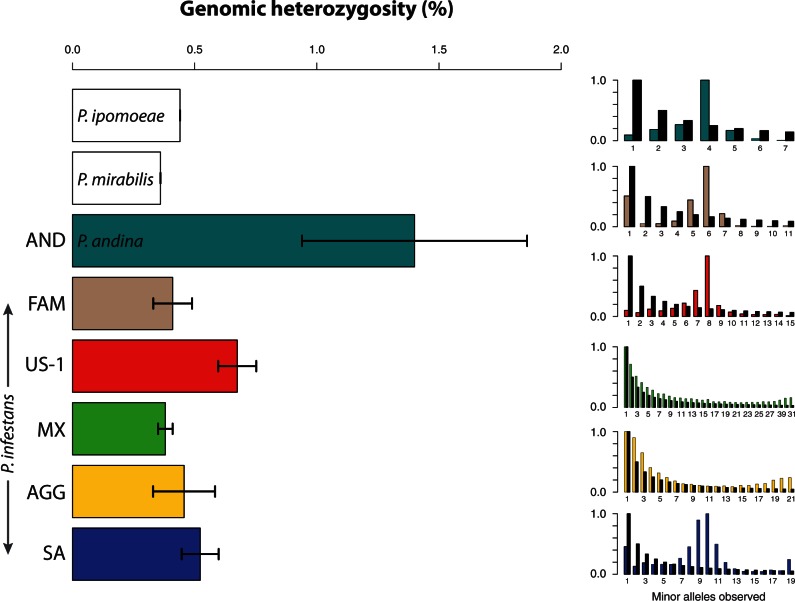
Heterozygosity mirrors reproductive strategy within *Phytophthora infestans* and *P. andina*. Left: Genomic heterozygosity (mean ± SD) for each genetic cluster. Right: Site frequency spectrum (SFS) for each genetic cluster. The *x* axis position of each bar indicates the number of occurrences of the minor allele in that genetic cluster. The height of each colored bar indicates the normalized proportion of genomic SNPs with that number of observed minor alleles. Black bar heights indicate the expected distribution (1/*x*) for a sexually reproducing population with a long period of constant population size under the infinite sites mutation model. Bar labels and shading are consistent with figure 1*d*. Bars for completely fixed or unobserved minor alleles (those with counts of 0 or 2*n*, where *n* is the number of individuals assigned to the cluster) are not shown.

Relatively high heterozygosity at nuclear loci has been used as evidence for a recent hybrid origin for *P. andina* (Goss et al. 2011). Mean genomic heterozygosity for the sampled nuclear genomes of *P. infestans* ranged from approximately 0.38% to 0.7% (fig. 2). *P. andina* genomes were highly heterozygous, with a mean value of 1.40%, followed by the *P. infestans* clonal lineages US-1 (0.67%) and SA (0.52%). Least heterozygous were the AGG (0.46%), FAM (0.41%), and MX (0.38%) clusters. One measure of clonality in lineages is the proportion of variant sites at which the same heterozygous genotype is fixed in every sample from the same population cluster. This value was computed for all genetic clusters and was also elevated in the clonal lineages (supplementary fig. S8, Supplementary Material online).

We also estimated linkage disequilibrium within our genetic clusters as an indicator of recent sexual reproduction (supplementary fig. S9, Supplementary Material online). Linkage decay in MX and AGG was consistent with sexual reproduction, whereas the FAM and AND clusters stood out in that they both showed complete linkage at all inter-SNP (single nucleotide polymorphism) distances, indicating complete clonality of these lineages.

The site frequency spectrum (SFS) not only captures past demographic changes but also appears to indicate clonality in certain populations (fig. 2). Although the MX and AGG populations showed distributions consistent with a long period of constant population size, the AND, FAM, US-1, and SA populations showed a major increase of genomic SNPs with allele frequencies around 50%. We tested whether mapping errors could produce the unusual SFS we observed in clonal lineages, but found that the pattern persisted even when limiting the analysis to uniquely mappable regions of the genome (supplementary fig. S10, Supplementary Material online). We propose that this SFS pattern signifies a long period of clonal reproduction that began when the original genotype lost the capacity and/or opportunity for sexual reproduction, fixing its own heterozygous alleles, which persisted at a 1:1 ratio within the clonal population formed by its descendants. Thereafter, deviation from 50% allele frequency at these sites would be facilitated only by forces such as genetic drift, selection, and mitotic recombination, which could drive some alleles to fixation in different clonal sublineages. Another possible explanation for these SFS observations is that the clonal lineages originated in hybridization events between divergent taxa, with a similar series of subsequent events.

Our investigation of loss-of-heterozygosity tracts revealed that they are relatively common throughout all clonal lineages examined. Notably, we estimate up to 15% of the genome in clonal lineages has been converted to homozygosity due to mitotic recombination (supplementary table S3, Supplementary Material online). The MX and AGG populations produced much smaller measures around 1%. From our estimates on supercontig1, we project that the total fraction of the genome phased by mitotic recombination is 6.1% for the SA lineage, 7.5% for the US-1 lineage, 9.6% for the FAM lineage, and 14.7% for the AND lineage.

### Phylogenomic Estimates from Phased Nuclear Haplotypes

Our previous analyses indicated substantial interhaplotype divergence in clonal individuals that could have implications for the nature of the most basal *P. infestans* lineages. To further explore the evolutionary history of the highly heterozygous clonal lineages, we performed local-scale, haplotype frequency-based phasing on the genotype calls for 55 ingroup nuclear genomes. Assessing this phasing using publicly available Sanger sequences from corresponding cloned PCR products, we estimated the error rate at approximately 1.6% (supplementary table S4, Supplementary Material online). We then used the resulting 110 haplotype sequences to infer phylogenies of individual supercontigs (supplementary figs. S11–S18, Supplementary Material online). Remarkably, these phylogenetic trees yield insight into the mode of reproduction of some lineages. In a sexually reproducing population, sequence divergence that accumulates on chromosome copies is shared among individuals through meiotic recombination, but we observed a possible “Meselson effect” in the haplotype sequences from known clonally reproducing lineages. As theorized by Birky (1996), in these samples two haplotype sequences from the same individual are more divergent from each other than they are from corresponding haplotype sequences of other individuals from the same clonal lineage.

**Fig. 3.**
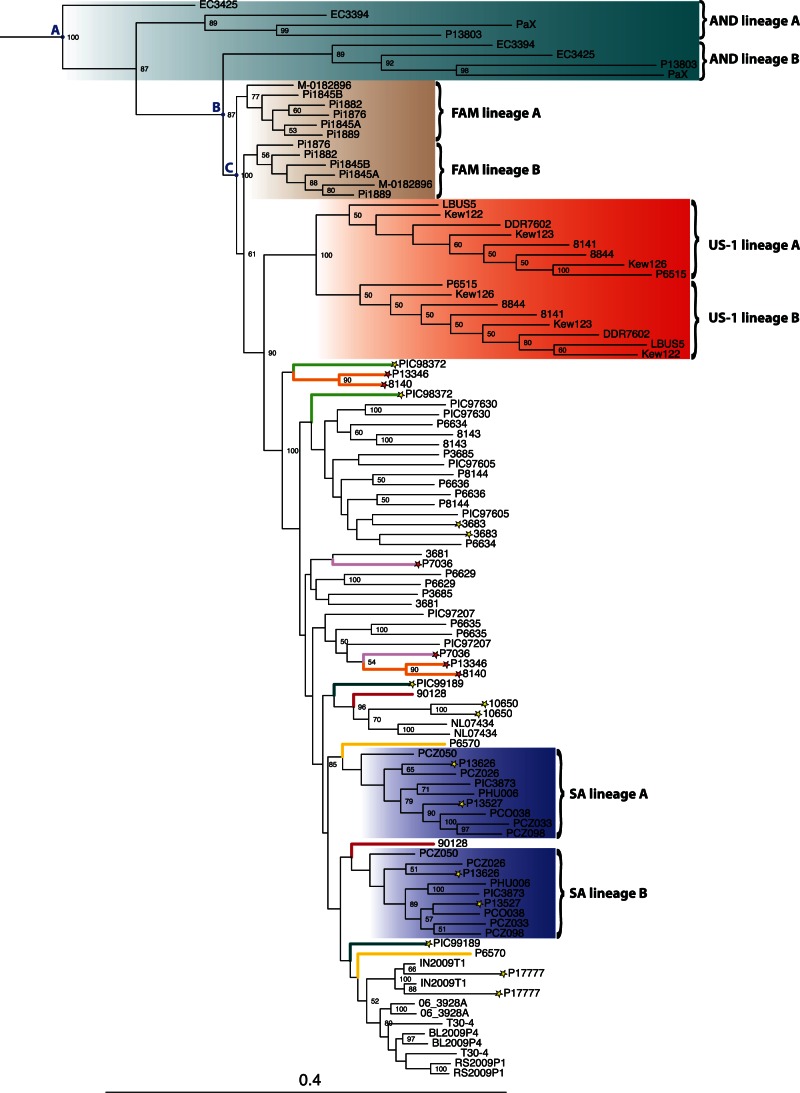
Maximum-likelihood phylogeny of phased haplotypes for supercontig 1, the largest scaffold in the T30-4 reference genome. For clarity, long branches leading to the outgroup sample haplotypes are not shown. Nodes A, B, and C are described in the text. Scale bar is given in nucleotide substitutions per site. Node labels indicate bootstrap support of 50% or greater. Major clonal lineages are shaded corresponding to figure 1*d*. Colored branches indicate other samples with large divergence between their haplotype sequences. Red stars, HERB-1 mtDNA. Yellow stars, non-*Solanum tuberosum* host.

Individual *P. andina* mitogenomes belong to divergent clades that suggest a hybrid origin from a *P. infestans* parent (fig. 1*b*). Similarly, *P. andina* nuclear genome haplotypes formed two deeply divergent clonal lineages for all supercontig trees examined. This divergence is apparently much older than the divergence between the *P. andina* haplotypes contributed by an ancient *P. infestans* parent and the rest of the *P. infestans* haplotypes observed (fig. 3). To approximate this age difference, we calculated the ratio of the total branch length between node A (MRCA of all *P. andina* haplotypes) and node C (MRCA of all *P. infestans* haplotypes) to the total branch length between node B (MRCA of all *P. infestans*-like haplotypes) and node A (supplementary table S5, Supplementary Material online). This ratio was calculated for the nine largest supercontigs, yielding a mean 6.1-fold older time of divergence from the ancestor of *P. infestans* for these two classes of *P. andina* haplotypes. Thus we argue that a non-*P. infestans* parent contributed one lineage of *P. andina* haplotypes, whereas the other lineage was contributed by a parent closely related to the root of all previously identified *P. infestans* haplotypes.

Haplotype sequences occupying the basal branches of clades containing isolates from the MX, AGG, and SA clusters often were apparently clonally evolving as evidenced by highly supported divergence between phased haplotypes from the same individual (supplementary figs. S11–S18, Supplementary Material online). For seven of the nine supercontigs examined, haplotype sequences belonging to isolates collected from non-*Solanum tuberosum* hosts were positioned in groups that were basal to the rest of MX, AGG, and SA isolates. These relatively well-supported divergence patterns were different for each supercontig examined. For example, for supercontigs 1, 4 and 5, one haplotype from isolate P98372 (collected from *S. demissum*) is basal to all other MX-cluster sequences. At supercontig 8, one haplotype from isolate PIC99189 (collected from *S. stoloniferum*) is basal to all MX-cluster sequences. In the highest-likelihood phylogenetic trees of the five remaining supercontigs, basal or nearly basal positions were occupied by groups of wild-host MX isolates or MX isolates harboring the HERB-1 mitogenomic lineage. These results were further supported by Shimodaira–Hasegawa (SH) topology tests. For each supercontig phylogenetic analysis, SH testing identified between 0 and 21 equally likely trees (supplementary table S6, Supplementary Material online). In every case in which equally likely trees were identified, 100% of the equal-likelihood trees supported the node segregating a basal group containing wild-host MX isolates or MX isolates harboring the HERB-1 mitogenomic lineage.

### Genetic Differentiation and Gene Flow

**Fig. 4.**
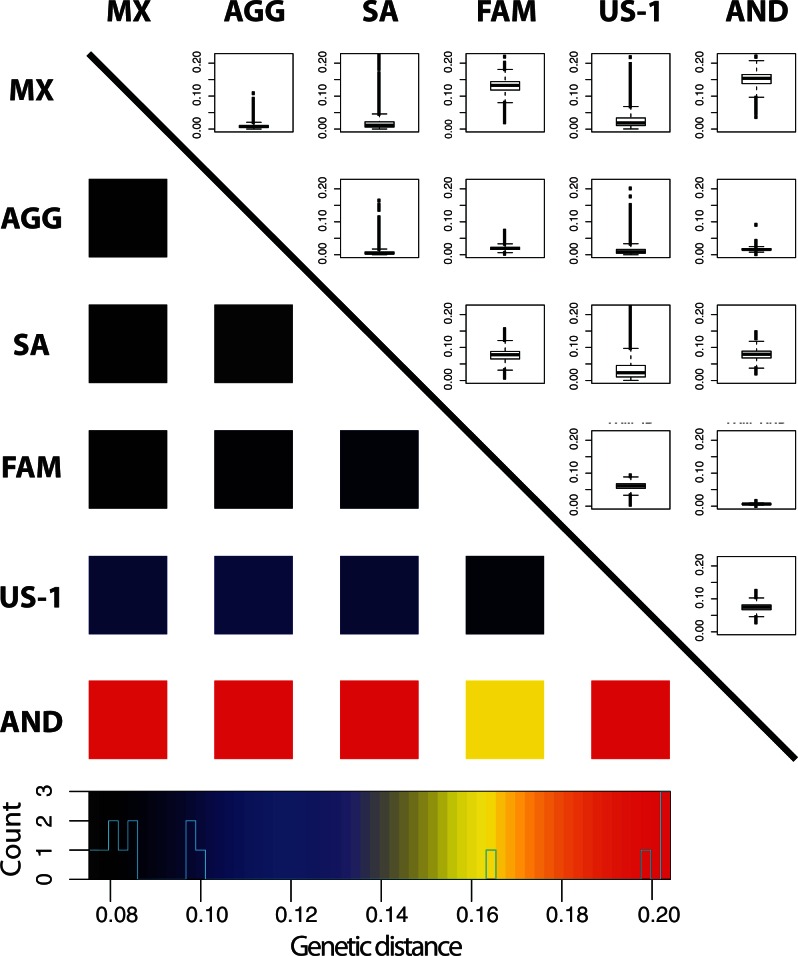
Pairwise measures of population differentiation and genetic distance between populations of *Phytophthora infestans* and *P. andina*. Upper: Genomic F_ST_ estimates displayed in boxplots. Box height indicates the interquartile range. Whiskers extend to 1.5 the interquartile range. Bold line indicates the median value. Lower: Heatmap of genetic distance estimates. Histogram over color legend displays the number of measurements within each genetic distance bin.

The *P. infestans*-like haplotype lineage preserved within *P. andina* genomes may be a relic of ancient gene flow that provides insight into the evolutionary history of *P. infestans* in the New World. To evaluate the connectedness of our defined groups, we quantified intercluster differentiation using mean pairwise genetic distances (supplementary table S7, Supplementary Material online) and the distribution of population differentiation (F_ST_) in pairwise comparisons (fig. 4 and supplementary table S8, Supplementary Material online). The smallest distance to the AND cluster is from FAM, whereas the lowest pairwise F_ST_ value for the FAM cluster is with AND, indicating a close relationship between these lineages. Very small distances between the MX, AGG, SA, and FAM clusters suggest close genetic relationships between them. Our measures of F_ST_ revealed that the AGG population cluster is by far the least differentiated on average, likely due to a history of admixture and gene flow with both Mexican and South American genotypes.

The measures establish a strong genetic relationship between *P. andina* and the FAM cluster that suggests some ancient gene flow. To gain insight into any putative past hybridization between *P. andina* and *P. infestans* populations, we looked for archaic admixture between our genetic clusters of interest using the *D*-statistic, originally developed to measure introgression of the Neanderthal genome into modern humans (Green et al. 2010). Population-wise *D*-statistics (Durand et al. 2011) show that FAM specimen genomes contain strong signals of introgression with AND (supplementary table S9, Supplementary Material online). These results were consistent no matter which *P. infestans* population was chosen for comparison (P_2_). US-1 showed much smaller but significant values of introgression with *P. andina*. We detected no introgression of *P. andina* with the SA or MX genetic clusters; thus, *P. andina* has no history of introgression with our sample of modern *P. infestans* populations in Mexico or South America. We also detected no signal of introgression of FAM or US-1 historical outbreak specimen clusters into MX or SA clusters. Similarly, we detected no signal of introgression of MX or SA populations into the FAM cluster, indicating that historical outbreaks came from a source population that had not recombined with the Mexican or South American populations we examined in this study.

Phylogenetic trees have been widely used in population genetics to visualize relationships among populations. Although a bifurcating phylogeny can provide a valuable initial assessment, this model assumes that populations split and experienced no further interpopulation gene flow, which is not always the case. Past hybridization events, like those in this report, violate this basic assumption of a bifurcating tree and may result in misleading inferences. To account for this effect, we used TreeMix (Pickrell and Pritchard 2012) to infer patterns of population splitting and mixing from genome-wide allele frequency data within the previously defined genetic clusters.

**Fig. 5.**
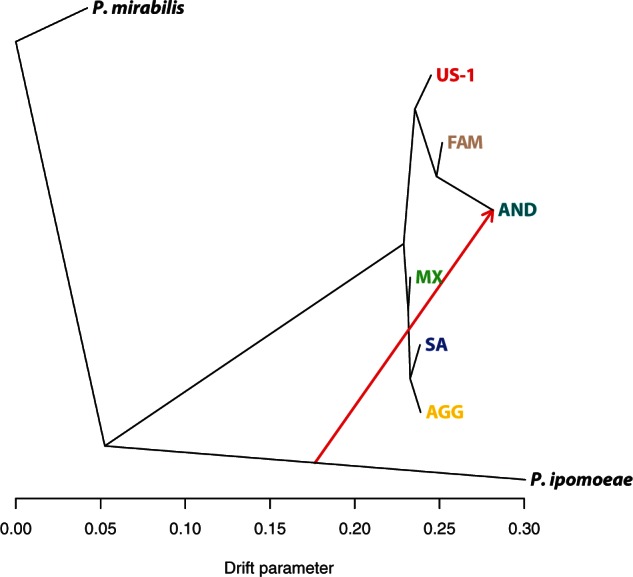
TreeMix graph representing population splitting patterns of the *Phytophthora* groups studied. *Phytophthora mirabilis* was used as outgroup and the length of the branches is proportional to the genetic drift of each population. Analysis with TreeMix reveals that *P. andina* resulted from a major hybridization event between an unknown outgroup related to *P. ipomoeae* and a *P. infestans* genotype most related to FAM, which caused the Irish potato famine. The arrow shows the direction of the primary inferred hybridization edge, and its color indicates a migration weight (the fraction of ancestry derived from the migration event) of 34%.

Under an assumption of zero gene flow events, the TreeMix model resulted in extremely high error residuals, indicating that our data are most compatible with one or more admixture events in the history of the *P. infestans* and *P. andina*, which is compatible with a complex history of human-mediated admixture (supplementary fig. S19, Supplementary Material online). Allowing for a single mixture event, we see strong gene flow (34% weight) from an ancestral *P. ipomoeae* population to the *P. andina* cluster AND, which is placed in a sister position relative to the FAM population (fig. 5). This primary event indicates an origin of *P. andina* as the result of hybridization between an unknown relative of *P. ipomoeae* and a close relative of the FAM clade, which has a recent common ancestor with *P. andina*. Assuming more than one interpopulation gene flow event, there are signs of gene flow at least between the US-1 cluster and the ancestor of both *P. andina* and the FAM clade. At 42.3% weight, this secondary event reflects a contribution of early US-1 populations to ancestors of the FAM lineage that caused the famine in Europe.

## Discussion

### Relating the Hybrid *P. andina* and the Irish Potato Famine

Our analyses provide strong evidence for a shared evolutionary history of *P. andina* and the *P. infestans* genotypes that triggered the first global outbreak of potato blight disease in 1845 and ultimately underpinned the catastrophic Irish potato famine. Specifically, genomic phasing of the sequence data from four modern isolates of the diploid hybrid *P. andina* recovers four haploid genome sequences of an ancient *P. infestans* lineage. Most notably, this lineage is sister to all modern and historic *P. infestans* genomes that have been sequenced so far, and so predates the famine-era lineage introduced outside the New World in the 1840s. We acknowledge, however, that one likely effect of the estimated 1.6% phasing switch error rate on the phylogenetic position of the *P. andina* would be to attract the branches of *P. infestans*-like haplotypes toward outgroup branches. Thus although we cannot be entirely certain of the precise placement of these *P. andina* haplotypes, the low error rate and the other evidence we present suggest that they belong within the FAM lineage. It is also unknown if this lineage still persists in nature. Still only a small number *P. infestans* and *P. andina* genomes have been sequenced, so more intense sampling in Mexico and South America, followed by additional genomic sequencing, could settle the question.

*Phytophthora andina* is thought to have originated from multiple hybridizations involving *P. infestans* (Blair et al. 2012). Our measures of exceptionally high genomic heterozygosity confirm a hybrid origin of diploid *P. andina* nuclear genomes, as previously inferred from a few nuclear markers (Goss et al. 2011). Owing to the unprecedented number and diversity of genomes analyzed, our study now provides insights into the nature of these hybridization events and important clues about when and where they occurred. Previous studies determined that isolates of *P. andina* possess highly divergent Ic or Ia mtDNA haplotypes (Adler et al. 2004; Oliva et al. 2010; Goss et al. 2011). However, our tip-calibrated mitogenome phylogeny indicates that type-Ia *P. andina* isolates actually carry the HERB-1 lineage in Ecuador. The Bayesian estimate of the mitochondrial tree clearly reveals at least two divergent mitochondrial lineages among the 19th-century mitogenomes. Indeed, as the Ia *P. andina* sequences cluster with relatively high support within different clades of the HERB-1 lineage, it seems that there were at least two hybridization events in which the maternal lineage that gave rise to *P. andina* was drawn from the same pool of *P. infestans* diversity that was introduced to 19th-century Europe. As neither of these *P. andina* mitogenomes diverged earlier than the MRCA of all historical HERB-1 *P. infestans* samples, this contradicts the hypothesis that a single mitochondrial haplotype was introduced into Europe in the 19th century (Yoshida et al. 2013). Instead, as suggested by Martin et al. (2014), at least two HERB-1 haplotypes were introduced.

Hybridization between the two parent species of *P. andina* must have occurred where they shared a host range. Both TreeMix analysis and *D*-statistics support a signal of ancestral admixture between *P. andina* isolates and the historical FAM samples. This is conclusive evidence that FAM-like genotypes once existed in Ecuador or Peru, the known range of *P. andina*. However, despite the fact that *P. andina* and *P. infestans* share common hosts in South America, and likely have had opportunities to mate and produce pathogenic offspring (Adler et al. 2004; Pérez 2009; Forbes et al. 2013), *D*-statistics indicate no introgression between *P. andina* and our samples of the SA cluster, which were largely collected from modern South American populations. This suggests that *P. andina* has reproduced asexually since its initial formation, or that it has always been incompatible with *P. infestans*. Similarly, we did not detect significant introgression between the FAM or US-1 groups and the MX or SA clusters. If sexual reproduction still occurred in the ancestors of these lineages after divergence of the MX and SA clusters, then our results suggest that the FAM and US-1 lineages either originated outside our sampled geographic range or were formerly segregated from other *P. infestans* populations on a non-*S. tuberosum* host.

### Allele Sequence Divergence within Clonal Lineages

In partially clonal *P. infestans* genomes, the differential accrual of mutations on sister chromosomes may produce a positive correlation between genomic heterozygosity and the time since last sexual reproduction. The MX and AGG isolates have the lowest levels of genomic heterozygosity as well as LD decay that indicates recent sexual recombination. Sexual reproduction is known to occur in modern populations of *P. infestans* in North Europe, but United States lineages are mostly asexual (Fry et al. 2015). Although the low heterozygosity of herbarium-derived FAM clonal lineage samples is closer to the mean heterozygosity of the MX and AGG samples than to the clonal SA samples, this is probably an effect of “frozen” evolution in this lineage, which may have only lost sexual reproduction not long before the collection date. In contrast, chromosomes within the US-1 samples, which were collected more recently, have had more time to accrue mutations and increase heterozygosity. We ascribe the especially high heterozygosity of the *P. andina* isolates to both the accrual of different mutations on sister chromosomes since the loss of sexual reproduction as well as their divergent hybrid chromosomes. We cannot rule out, however, that genomic heterozygosity in clonal lineages is mostly generated during an initially wide cross between divergent genotypes, after which sexual reproduction is lost.

### The Geographic Origin of *P. infestans*

Our data also allow us to readdress the geographic origin of *P. infestans*. Our date estimates for the speciation of both *P. infestans* and *P. andina* do not preclude a human connection, as they are well within the time of Incan trade and early European exploration of Central and South America. Thus, it is tantalizing to speculate that both species originated from human-mediated dispersal/hybridization events from the Andean region to Mexico. Although it has been recently claimed that *P. infestans* originated in Mexico (Yoshida et al. 2013; Goss et al. 2014), our results demonstrate that nuclear genome lineages basal to modern *P. infestans* diversity exclusively consist of samples collected outside Mexico. With its characteristic and deep split between type I and predominantly Andean type II haplotypes, the mitogenome phylogeny of *P. infestans* also suggests an ancient segregation event that cannot satisfactorily be explained by an entirely Mexican origin of the species (Griffith and Shaw 1998; Avila-Adame et al. 2006; Gómez-Alpizar et al. 2008; Yoshida et al. 2013; Martin et al. 2014). Indeed, because the most basal *P. infestans*-like nDNA haplotypes survive within *P. andina*, found only in the highlands of Peru and Ecuador, our phylogenetic analyses support the species’ center of origin there.

### Sampling Bias and Early Host Switching in Mexican Populations

Our observation that the FAM lineage is sister to all modern nuclear genomic diversity was unexpected. Our nuclear genome phylogeny differs substantially from those of Martin et al. (2013) and Yoshida et al. (2013), both of which reconstructed trees using genomic sequence data from small numbers of samples. Our larger sample captures more completely *P. infestans* diversity, which we feel enables a more accurate reconstruction of the genealogy of the included genomes. However, phylogenetic reconstruction can be influenced by the inclusion of lineages that have undergone appreciable genetic exchange (e.g., Larson et al. 2012). Specifically, recombination can lead to an apparent reduction in the time to the MRCA (Schierup and Hein 2000), which would favor the grouping of the sexually reproducing lineages that exchanged genetic material more recently. Clonal lineages would then appear genetically distinct from sexually reproducing lineages. This is not likely in our case since the genetic distance from the FAM lineage to all other modern groups is approximately equal, suggesting that none of these groups is the source population of the FAM lineage, but that in fact, as can be interpreted from the phylogeny, the FAM lineage diverged early on from the lineage that gave rise to genotypes from contemporary *P. infestans* populations.

Clonal lineages at the most basal branches of the nuclear genome phylogeny suggest that the full extent of *P. infestans* diversity may not have been included in our analysis. But the only apparent bias in our selection of available isolates, which span known *P. infestans* diversity, is that our *P. infestans* samples were primarily collected from *S. tuberosum* hosts. Most studies of the genetic diversity of *P. infestans* focus on isolates from potato and tomato crops due to their economic importance, but much “paleoendemic” diversity may exist on other Solanaceae of the Ecuadorian Andes (Adler et al. 2004). Thus an entirely different type of sampling bias could explain our results, and even more ancestral lineages may exist on unsampled wild *Solanum* hosts. We conclude that full resolution of the evolutionary history of *Phytophthora* species in the Ic clade necessitates intensive sampling of all the clade 1c species over a broader geographic area that includes Central and South America.

In our phylogenetic analyses, the relative position of basal-tending Mexican isolates was often inconsistent between the mito-/nuclear genome phylogenies. Unsurprisingly, these clonal lineages tend to carry HERB-1 mtDNA, which we link with ambiguous geographic origins and nuclear/mitochondrial phylogenetic incongruence. These observations could be related to the stochastic nature of mitochondrial genome inheritance, but are also consistent with exceptional events such interspecific or interlineage hybridization (Rieseberg et al. 1996) in the early evolution of these lineages. More curious are observations within Mexican populations of mosaic nuclear genomes with inconsistent signals of ancestry across different genomic blocks, which may best be explained as an effect of partial hybridization (unequal inheritance of parental markers) that is common in *Phytophthora* (Förster and Coffey 1990; Pérez 2009). Together, these findings point to a possible origin of modern Mexican *P. infestans* populations through jumps to domestic potato from multiple wild *Solanum* hosts, such as *S. demissum*, which is distributed from northern Mexico to Guatemala and was first collected shortly after the first global outbreak as source material for resistance breeding in European potato cultivars (Lindley 1848).

### Alternative Scenarios for the Origin of P. infestans

Synthesizing our new findings with historical evidence that *P. infestans* first appeared in Europe shortly after its first observations in the United States enables us to propose two scenarios for the origin of *P. infestans* and its subsequent expansion out of the Americas into Europe (Bourke 1991). In the first, favored by Goss et al. (2014), *P. infestans* originated in Mexico, and sexual reproduction is the ancestral state of the species. The FAM/HERB-1 lineage was formerly at high frequency in Mexico, and Mexican FAM genotypes were transported by humans almost simultaneously north to the United States and south to the Ecuadorian Andes. From the United States, a few of these genotypes were further transported to Europe in the early 1840s. In Ecuador, the FAM-like individual lived in sympatry with as-yet-undiscovered clade 1c *Phytophthora* species on *S. betaceum* or another solanaceous host, where they formed the offspring we now know as *P. andina* through multiple hybridization events. The HERB-1 mitogenomic lineage persists to this day in Mexico, but local demographic fluctuations there have left it as only a relic on potato populations, whereas Mexican FAM genotypes have since been completely outcompeted and replaced.

In the second scenario, FAM genotypes long existed in South America possibly on nonpotato hosts, where they lived in sympatry with an unknown clade 1c *Phytophthora* species and with which they formed hybrids on multiple occasions. South American clonal lineages were introduced to Mexican domesticated potatoes, possibly through a host jump, and FAM genotypes from South America were also introduced to the United States and then Europe. The FAM lineage may persist unsampled on solanaceous hosts in Ecuador and Peru, but more recent introductions of other clonal lineages from Mexico now dominate these potato populations.

### Toward Global Population Genomics of Complex Pathogens

Migration and population substructure (e.g., on different hosts) are some of the major drivers of *Phytophthora* evolution (Flier et al. 2003; Goss et al. 2009), but are also problematic for elucidating the recent evolutionary history of these species. Mosaic genomes appear to be the rule for *P. infestans* and may be best explained by complex host switching and recombination in the earliest history of *P. infestans* and *P. andina*. Much promise lies in the coming flood of genomic data from pathogen populations undergoing complex biological processes, such as hybridization and occasional clonal reproduction (partial clonality; e.g., Arnaud-Haond et al. 2014). Data sets like ours show that multiple lines of evidence generated from sophisticated genomic toolsets can help to elucidate ancestry and recent divergence of fast-evolving species like these, and ultimately will provide opportunities to study host-specific adaptation and the evolution of resistance loci in great detail. Our work highlights the importance of disparate live culture and herbarium collections for future genomic studies of *P. infestans*, as well as the necessity of their careful curation.

## Materials and Methods

### Sample Sources and DNA Extraction

Cryostorage-preserved *P. infestans* isolate mycelium and DNA from historic herbarium samples were obtained from J.B.R.’s research collection, and more recent samples purchased from Mike Coffey’s “World *Phytophthora* Collection” (supplementary table S1, Supplementary Material online). Genomic DNA was extracted from mycelium using two protocols described in detail previously (Martin et al. 2013, 2014). DNA was extracted from *P. andina* isolate EC3394 using a cetrimonium bromide (CTAB) procedure as in Ristaino et al. (2001). To conduct a combined analysis of all available genomic data along with the *Phytophthora* spp. genomes sequenced here, publicly available Illumina genomic shotgun sequence reads from previously sequenced *Phytophthora* spp. modern isolates and historical samples were obtained from the Sequence Read Archive public database under accession codes ERP003267, ERP002419, ERP002420, ERP002550, and ERP002552 and from the European Nucleotide Archive under accession code PRJNA52431.

### Bayesian Phylogenetic Analysis of Mitogenome Sequences

To estimate the evolutionary timescale of *P. infestans*, we performed a Bayesian dating analysis on an alignment of iteratively assembled mitochondrial genome sequences (supplementary file S1, Supplementary Material online) using BEAST v1.8.0 (Drummond et al. 2012). Missing nucleotides and gaps were treated as ambiguous bases (N). The Bayesian information criterion was used to identify the best-fitting substitution model for each of five data subsets (three codon positions of protein-coding genes, rRNA genes, and intergenic sites). We used the flexible skyride coalescent prior for the tree (Minin et al. 2008). An initial analysis using an uncorrelated lognormal relaxed clock model (Drummond et al. 2006) indicated a very low degree of rate heterogeneity across branches, so we used a strict clock model. The sampling times of the sequences were used to calibrate the estimates of the substitution rate and coalescence times. The ages of the sequences were previously shown to be sufficient for calibration, based on a date-randomization test (Martin et al. 2014). Two independent Markov chain Monte Carlo analyses were performed to estimate the posterior distributions of parameters, including the tree.

### Creation of a Multiple Sequence Alignment of the Nuclear Genome

The GATK UnifiedGenotyper tool was used with a minimum genotype quality score of 30 to produce Variant Call Format (VCF) files, from which a multiple sequence alignment (MSA) of the 57 nuclear genome sequences was populated with polymorphic bases using custom Python scripts. In this VCF to MSA format conversion, heterozygous positions were assigned standard IUPAC nucleotide ambiguity codes, and insertion/deletion variants were ignored. Included sites were conservatively restricted to those uniquely mappable to the *P. infestans* T30-4 reference as determined by the GEnome Multitool (GEM) mappability tool (Derrien et al. 2012), using a *k*-mer size of 65 bp and maximum of three mismatches. These uniquely mappable regions of the genome amounted to 66.7 Mb. This master nDNA SNP alignment was filtered for various values of minimum read depth in downstream analyses.

### Maximum-Likelihood Phylogenetic Analysis of Nuclear Genomes

Phylogenetic inference was performed using maximum likelihood (ML) with RAxML v7.3.5 (Stamatakis 2006) on two data sets: 1) Concatenated alignments of diploid genotypes and 2) phased haplotype sequences. All RAxML analyses employed the GTRGAMMA (General Time Reversible with the Γ model of among-site rate heterogeneity and four discrete rate categories) substitution model (Yang 1996). Consensus trees were created by drawing node support from 100 bootstrap replicates on the tree of highest likelihood. Each tree was rooted using *P. mirabilis* sequence as outgroup.

For the genotype-based analysis, master nDNA SNP alignment positions with a minimum depth of two reads were considered. To reduce computational burden, only the six largest supercontigs (comprising 861,892 SNPs) were used. For analysis of phased haplotypes, BEAGLE v3.32 (Browning 2006; Browning SR and Browning BL 2007) utilized ANGSD genotype likelihoods under default settings to infer two phased haplotype sequences and impute missing genotype calls for each reference supercontig. As BEAGLE’s power to determine and impute haplotypes depends on larger sample sizes, in our analysis all individuals from all species were phased together. Only SNP positions with allelic *r*^2 ^≥ 0.8 were included, but applying various thresholds for this parameter produced virtually identical phasing results. For each of the nine longest supercontigs, we used RAxML as described above to infer a phylogeny from an MSA of all haplotype sequences (2 × 57 sequences).

To rigorously assess the phylogenetic placement of seemingly basal clonal lineages collected from non-*S. tuberosum* hosts, we used RAxML to perform the nonparametric SH test of topology (Shimodaira and Hasegawa 1999). For the phylogenetic analysis of each supercontig sequence alignment, this test identified any bootstrap replicate tree whose likelihood was not significantly (at the 1% level) lower than that of the highest-likelihood tree. We then used RAxML to calculate the proportion of these equally likely trees that supported each of the nodes of the highest-likelihood tree.

### Bayesian Dating Analysis of Nuclear Genes

To estimate the evolutionary timescale of the nuclear genome, we used a Bayesian phylogenetic approach. Owing to the effects of recombination, different loci are likely to have incongruent genealogies, including unequal coalescence times. Accordingly, we performed a separate dating analysis of each gene and summarized the gene-specific date estimates to obtain a distribution of coalescence times. To perform the dating analysis, we limited the data set to 50 genome sequences with known collection dates. Coding sequences for each of 18,178 annotated gene and pseudogene sequences were extracted from all 57 samples and concatenated into an MSA of each gene’s coding regions. We only used data from the third codon positions to minimize the confounding effects of selection on estimates of recent evolutionary events (Ho et al. 2011). To reduce the impacts of missing data (e.g., Lemmon et al. 2009), we only retained sites with data available in greater than 50% of the samples. After these steps, we excluded any genes with fewer than 500 aligned sites. This left a total of 3,676 genes for the dating analysis (supplementary file S2, Supplementary Material online). We used BEAST 1.8.0 to estimate the genealogy from each gene, with the timescale calibrated using the ages of the sequences. We used the HKY+G substitution model, representing a balance between model complexity and biological plausibility. A strict clock was assumed for all genes. Markov chain Monte Carlo analyses were run in duplicate to check for convergence.

### Admixture Proportion and Genetic Differentiation Estimation

ANGSD v0.588 (Nielsen et al. 2012; Korneliussen et al. 2013) was used to estimate genotype likelihoods for each sample at sites likely to contain SNP variants (command-line options *-GL 1 -doGlf 2 -SNP_pval 1e-6*), resulting in 1,371,306 SNP sites where genotypes passed the confidence filter in at least 14 (25%) of the individuals. We then employed NGSadmix v32 (Skotte et al. 2013) to use these genotype likelihoods to estimate the proportion of each resequenced *Phytophthora* spp. genome that belonged to a varying number of assumed ancestral populations under the assumption of Hardy–Weinberg equilibrium (e.g., Pritchard et al. 2000). For each number of assumed ancestral populations (*K*) from 1 to 12, ten independent replicates of NGSadmix were performed to evaluate convergence of the simulations, and the replicate of highest likelihood was chosen. To estimate pairwise population genomic differentiation (*F*_ST_), we used the software package ngsTools (Fumagalli et al. 2013).

### TreeMix Analyses

We used TreeMix v1.12 to account for past hybridization events that violate the basic assumptions of a bifurcating phylogenetic tree. More specifically, genotypes were called from genotype likelihoods using ANGSD (command-line options *-GL 1 -SNP_pval 1e-6 -doGeno 5 -baq 1 -C 50 -minQ 10 -setMinDepth 50 -doPost 2 -postCutoff 0.5 -geno_minDepth 2*) and individual samples were merged into populations according to their assignments to the previously defined genetic clusters. Outgroup isolates PIC99167 (*P. ipomoeae*) and PIC99114 (*P. mirabilis*) were considered separate populations. After merging the genotype data, only biallelic variant sites without missing data were provided to TreeMix, which was run assuming from 0 to 5 migration events, with *P. mirabilis* as the root population (command-line option *-root*), and performing a round of global rearrangements of the graph after initial fitting (command-line option *-global*). To account for any linkage disequilibrium in sexual populations, we grouped together blocks of 1,000 SNPs. To avoid convergence to local maxima, we ran 100 replicates for each scenario, retaining only the replicate with the highest likelihood.

## Supplementary Material

Supplementary files S1 and S2, materials and methods, references, figures S1–S19, and tables S1–S9 are available at *Molecular Biology and Evolution* online (http://www.mbe.oxfordjournals.org/).

Supplementary Data

## References

[msv241-B1] Abad ZG Abad JA 1997 Another look at the origin of late blight of potatoes, tomatoes, and pear melon in the Andes of South America. Plant Dis. 81:682–688. 10.1094/PDIS.1997.81.6.682 30861859

[msv241-B2] Adler NE Chacón G Flier WG Forbes GA 2002 The Andean fruit crop, pear melon (*Solanum muricatum*) is a common host for A1 and A2 strains of *Phytophthora infestans* in Ecuador. Plant Pathol. 51:802.

[msv241-B3] Adler NE Erselius LJ Chacon MG Flier WG Ordonez ME 2004 Genetic diversity of *Phytophthora infestans* sensu lato in Ecuador provides new insight into the origin of this important plant pathogen. Phytopathology 94:154–162. 18943538 10.1094/PHYTO.2004.94.2.154

[msv241-B4] Arnaud-Haond S Moalic Y Barnabé C Ayala FJ Tibayrenc M 2014 Discriminating micropathogen lineages and their reticulate evolution through graph theory-based network analysis: the case of *Trypanosoma cruzi*, the agent of Chagas disease. PLoS One 9:e103213. 25148574 10.1371/journal.pone.0103213 PMC4141739

[msv241-B5] Avila-Adame C Gómez-Alpizar L Buell RC Ristaino JB 2006 Mitochondrial genome sequencing of the haplotypes of the Irish potato famine pathogen, *Phytophthora infestans*. Curr Genet. 49:39–46. 16328503 10.1007/s00294-005-0016-3

[msv241-B6] Balloux F Lehmann L de Meeûs T 2003 The population genetics of clonal and partially clonal diploids. Genetics 164:1635–1644. 12930767 10.1093/genetics/164.4.1635 PMC1462666

[msv241-B7] Berkeley MJ 1846 Observations, botanical and physiological on the potato murrain. J Hortic Soc Lond. 1:9–34.

[msv241-B8] Birch PRJ Cooke DEL 2013 The early days of late blight. eLife 2:e00954. 23795302 10.7554/eLife.00954 PMC3687336

[msv241-B9] Birky CW Jr 1996 Heterozygosity, heteromorphy, and phylogenetic trees in asexual eukaryotes. Genetics 144:427–437. 8878706 10.1093/genetics/144.1.427 PMC1207515

[msv241-B10] Blair JE Coffey MD Martin FN 2012 Species tree estimation for the late blight pathogen, *Phytophthora infestans*, and close relatives. PLoS One 7:e37003. 22615869 10.1371/journal.pone.0037003 PMC3355167

[msv241-B11] Bourke A 1991 Potato late blight in Europe in 1845: the scientific controversy. In: Lucas JA Shattock RD Shaw DS Cooke LR, editors*. *Phytophthora. New York: Cambridge University Press p. 12–24.

[msv241-B12] Bourke PMA 1964 Emergence of potato blight, 1843-46. Nature 203:805–808.

[msv241-B13] Brasier C 2000 The rise of the hybrid fungi. Nature 405:134–135. 10821256 10.1038/35012193

[msv241-B14] Browning SR 2006 Multilocus association mapping using variable-length Markov chains. Am J Hum Genet. 78:903–913. 16685642 10.1086/503876 PMC1474089

[msv241-B15] Browning SR Browning BL 2007 Rapid and accurate haplotype phasing and missing data inference for whole genome association studies using localized haplotype clustering. Am J Hum Genet. 81:1084–1097. 17924348 10.1086/521987 PMC2265661

[msv241-B16] Cárdenas M Grajales A Sierra R Rojas A González-Almario A Vargas A Marín M Fermín G Lagos LE Grünwald NJ 2011 Genetic diversity of *Phytophthora infestans* in the Northern Andean region. BMC Genet. 12:23. 21303555 10.1186/1471-2156-12-23 PMC3046917

[msv241-B17] Chamnanpunt J Shan WX Tyler BM 2001 High frequency mitotic gene conversion in genetic hybrids of the oomycete *Phytophthora sojae*. Proc Natl Acad Sci U S A. 98:14530–14535. 11724938 10.1073/pnas.251464498 PMC64716

[msv241-B18] De Bary M 1863 Du developement de quelques champignons parasites. Ann Sci Nat. 20:1–143.

[msv241-B19] Derrien T Estellé J Marco Sola S Knowles DG Raineri E Guigó R Ribeca P 2012 Fast computation and applications of genome mappability. PLoS One 7:e30377. 22276185 10.1371/journal.pone.0030377 PMC3261895

[msv241-B20] Douglas NA Manos PS 2007 Molecular phylogeny of Nyctaginaceae: taxonomy, biogeography, and characters associated with a radiation of xerophytic genera in North America. Am J Bot. 94:856–872. 21636455 10.3732/ajb.94.5.856

[msv241-B21] Drenth A Goodwin SB Fry WE Davidse LC 1993 Genotypic diversity of *Phytophthora infestans* in the Netherlands revealed by DNA polymorphisms. Phytopathology 83:1087–1092.

[msv241-B22] Drummond AJ Ho SYW Phillips MJ Rambaut A 2006 Relaxed phylogenetics and dating with confidence. PLoS Biol. 4:e88. 16683862 10.1371/journal.pbio.0040088 PMC1395354

[msv241-B23] Drummond AJ Suchard MA Xie D Rambaut A 2012 Bayesian phylogenetics with BEAUti and the BEAST 1.7. Mol Biol Evol. 29:1969–1973. 22367748 10.1093/molbev/mss075 PMC3408070

[msv241-B24] Durand EY Patterson N Reich D Slatkin M 2011 Testing for recent admixture between closely related populations. Mol Biol Evol. 28:2239–2252. 21325092 10.1093/molbev/msr048 PMC3144383

[msv241-B25] Erwin DC Ribeiro OK 1996 *Phytophthora* diseases worldwide. St. Paul (MN): American Phytopathological Society Press.

[msv241-B26] Flier WG Grünwald NJ, Kroon LPNM, Sturbaum AK, van den Bosch TBM, Garay-Serrano E, Lozoya-Saldaña H, Fry WE, Turkensteen LJ 2003 The population structure of *Phytophthora infestans* from the Toluca Valley of central Mexico suggests genetic differentiation between populations from cultivated potato and wild *Solanum* spp. Phytopathology 93:382–390. 18944351 10.1094/PHYTO.2003.93.4.382

[msv241-B27] Forbes GA Morales JG, Restrepo S, Pérez W, Gamboa S, Ruiz R, Cedeño L, Fermin G, Andreu AB, Acuña I, 2013 *Phytophthora infestans* and *Phytophthora andina* on Solanaceous hosts in South America. In: Lamour K, editor. *Phytophthora:* a global perspective. Oxfordshire (United Kingdom): CAB International p. 48–58.

[msv241-B28] Forbes GA Ristaino J Oliva RF Flier W 2011 A rebuttal to the letter to the editor concerning: defining species boundaries in the genus *Phytophthora*: the case of *Phytophthora andina*. Plant Pathol. 61:221–223.

[msv241-B29] Förster H Coffey MD 1990 Mating behavior of *Phytophthora parasitica*: evidence for sexual recombination in oospores using DNA restriction fragment polymorphisms as genetic markers. Exp Mycol. 14:351–359.

[msv241-B30] Fry W 2008 *Phytophthora infestans*: the plant (and R gene) destroyer. Mol Plant Pathol. 9:385–402. 18705878 10.1111/j.1364-3703.2007.00465.x PMC6640234

[msv241-B31] Fry WE Birch PRJ, Judelson HS, Grünwald NJ, Danies G, Everts KL, Gevens AJ, Gugino BK, Johnson DA, Johnson SB, 2015 Five reasons to consider *Phytophthora infestans* a reemerging pathogen*.* Phytopathology 105:966–981. 25760519 10.1094/PHYTO-01-15-0005-FI

[msv241-B32] Fumagalli M Vieira FG, Korneliussen TS, Linderoth T, Huerta-Sanchez E, Albrechtsen A, Nielsen R 2013 Quantifying population genetic differentiation from next-generation sequencing data. Genetics 195:979–992. 23979584 10.1534/genetics.113.154740 PMC3813878

[msv241-B33] Gómez-Alpizar L Carbone I Ristaino JB 2007 An Andean origin of *Phytophthora infestans* inferred from mitochondrial and nuclear gene genealogies. Proc Natl Acad Sci U S A. 104:3306–3311. 17360643 10.1073/pnas.0611479104 PMC1805513

[msv241-B34] Gómez-Alpizar L Hu C-H Oliva R Forbes G Ristaino JB 2008 Phylogenetic relationships of *Phytophthora andina*, a new species from the highlands of Ecuador that is closely related to the Irish potato famine pathogen *Phytophthora infestans*. Mycologia 100:590–602. 18833752 10.3852/07-074r1

[msv241-B35] Goodwin SB 1997 The population genetics of *Phytophthora*. Phytopathology 87:462–473. 18945128 10.1094/PHYTO.1997.87.4.462

[msv241-B36] Goodwin SB Fry WE 1994 Genetic analyses of interspecific hybrids between *Phytophthora infestans* and *Phytophthora mirabilis*. Exp Mycol. 18:20–32.

[msv241-B37] Goss EM Carbone I Grünwald NJ 2009 Ancient isolation and independent evolution of the three clonal lineages of the exotic sudden oak death pathogen *Phytophthora ramorum*. Mol Ecol. 18:1161–1174. 19222751 10.1111/j.1365-294X.2009.04089.x

[msv241-B38] Goss EM Cardenas ME, Myers K, Forbes GA, Fry WE, Restrepo S, Grünwald NJ 2011 The plant pathogen *Phytophthora andina* emerged via hybridization of an unknown *Phytophthora* species and the Irish potato famine pathogen, *P**. **infestans*. PLoS One 6:e24543. 21949727 10.1371/journal.pone.0024543 PMC3174952

[msv241-B39] Goss EM Tabima JF, DEL, Restrepo S, Fry WE, Forbes GA, Fieland VJ, Cardenas M, and NJ. Grünwald NJ 2014 The Irish potato famine pathogen *Phytophthora infestans* originated in central Mexico rather than the Andes. Proc Natl Acad Sci U S A. 11:8791–8796. 24889615 10.1073/pnas.1401884111 PMC4066499

[msv241-B40] Green RE Krause J, Briggs AW, Maricic T, Stenzel U, Kircher M, Patterson N, Li H, Zhai W, Fritz MH-Y, 2010 A draft sequence of the Neandertal genome. Science 328:710–722. 20448178 10.1126/science.1188021 PMC5100745

[msv241-B41] Griffith GW Shaw DS 1998 Polymorphisms in *Phytophthora infestans*: four mitochondrial haplotypes are detected after PCR amplification of DNA from pure cultures or from host lesions. Appl Environ Microbiol. 64:4007–4014. 9758833 10.1128/aem.64.10.4007-4014.1998 PMC106592

[msv241-B42] Grünwald NJ Flier WG 2005 The biology of *Phytophthora infestans* at its center of origin. Annu Rev Phytopathol. 43:171–190. 16078881 10.1146/annurev.phyto.43.040204.135906

[msv241-B43] Guha Roy S Grünwald NJ 2015 The plant destroyer genus *Phytophthora* in the 21st century. In: Chakraborty BN Thakore BBL, editors. Review of plant pathology. Vol. 6. Jodhpur (India): Scientific Publishers p. 387–412.

[msv241-B44] Haas BJ Kamoun S, Zody MC, Jiang RHY, Handsaker RE, Cano LM, Grabherr M, Chinnappa D. Kodira CD, Raffaele S, Torto-Alalibo T, 2009 Genome sequence and analysis of the Irish potato famine pathogen *Phytophthora infestans*. Nature 461:393–398. 19741609 10.1038/nature08358

[msv241-B45] Hansen EM Reeser PW Sutton W 2012 *Phytophthora* beyond agriculture. Annu Rev Phytopathol. 50:359–378. 22681450 10.1146/annurev-phyto-081211-172946

[msv241-B46] Ho SYW Lanfear R Bromham L Phillips MJ Soubrier J Rodrigo AG Cooper A 2011 Time-dependent rates of molecular evolution. Mol Ecol. 20:3087–3101. 21740474 10.1111/j.1365-294X.2011.05178.x

[msv241-B47] Korneliussen TS Moltke I Albrechtsen A Nielsen R 2013 Calculation of Tajima’s D and other neutrality test statistics from low depth next-generation sequencing data. BMC Bioinformatics 14:289. 24088262 10.1186/1471-2105-14-289 PMC4015034

[msv241-B48] Kroon LPNM 2010 The genus *Phytophthora*: phylogeny, speciation and host specificity [Ph.D. thesis]. [Wageningen (The Netherlands)]: Wageningen University.

[msv241-B49] Larson G Karlsson EK, Perria A, Webster MT, Ho SYW, Peters J, Stahl PW, Piper PJ, Lingaas F, Fredholm M, 2012 Rethinking dog domestication by integrating genetics, archeology, and biogeography. Proc Natl Acad Sci U S A. 109:8878–8883. 22615366 10.1073/pnas.1203005109 PMC3384140

[msv241-B50] Lassiter ES Russ C, Nusbaum C, Zeng Q, Saville AC, Olarte RA, Carbone I, Hu C-H, Seguin-Orlando A, Samaniego JA, 2015 Mitochondrial genome sequences reveal evolutionary relationships of the *Phytophthora* 1c clade species. Curr Genet. doi: 10.1007/s00294-015-0480-3. 10.1007/s00294-015-0480-3 PMC4659649 25754775

[msv241-B51] Lemmon AR Brown JM Stanger-Hall K Moriarty Lemmon E 2009 The effect of ambiguous data on phylogenetic estimates obtained by maximum likelihood and Bayesian inference. Syst Biol. 58:130–145. 20525573 10.1093/sysbio/syp017 PMC7539334

[msv241-B52] Lindley J 1848 Notes on the wild potato. R Hortic Soc J. 3:65–72.

[msv241-B53] Martin MD Cappellini E, Samaniego JA, Zepeda ML, Campos PF, Seguin-Orlando A, Wales N, Orlando L, Ho SYW, Dietrich FS, 2013 Reconstructing genome evolution in historic samples of the Irish potato famine pathogen. Nat Commun. 4; doi: 10.1038/ncomms3172. 10.1038/ncomms3172 PMC3759036 23863894

[msv241-B54] Martin MD Ho SYW Wales N Ristaino JB Gilbert MTP 2014 Persistence of the mitochondrial lineage responsible for the Irish potato famine in extant New World *Phytophthora infestans*. Mol Biol Evol. 31:1414–1420. 24577840 10.1093/molbev/msu086

[msv241-B55] May K Ristaino JB 2004 Identity of the mtDNA haplotype(s) of *Phytophthora infestans* in historical specimens from the Irish potato famine. Mycol Res. 108:471–479. 15229999 10.1017/s0953756204009876

[msv241-B56] Minin VN Bloomquist EW Suchard MA 2008 Smooth skyride through a rough skyline: Bayesian coalescent-based inference of population dynamics. Mol Biol Evol. 25:1459–1471. 18408232 10.1093/molbev/msn090 PMC3302198

[msv241-B57] Nielsen R Korneliussen T Albrechtsen A Li Y Wang J 2012 SNP Calling, genotype calling, and sample allele frequency estimation from new-generation sequencing data. PLoS One 7:e37558. 22911679 10.1371/journal.pone.0037558 PMC3404070

[msv241-B58] Oliva RF Chacón MG Cooke DE Lees AK Forbes GA 2007 Is *Phytophthora infestans* a good taxonomist? Host recognition in the *Phytophthora*/*Solanum* interaction. Acta Hortic. 745:465–471.

[msv241-B59] Oliva RF Kroon LPNM, Chacon G, Flier WG, Ristaino JB, Forbes GA 2010 *Phytophthora andina* sp. nov., a newly identified heterothallic pathogen of solanaceous hosts in the Andean highlands. Plant Pathol. 59:613–625.

[msv241-B60] Olmstead RG Bohs L, Migid HA, Santiago-Valentin E, Garcia VF, Collier SM 2008 A molecular phylogeny of the Solanaceae. Taxon 57:1159–1181.

[msv241-B61] Pérez RFO 2009 Occurrence of sympatric *Phytophthora* species in the highland of Ecuador [Ph.D. thesis]. [Zurich (Switzerland)]: Swiss Federal Institute of Technology.

[msv241-B62] Perez WG Gamboa JS, Falcon YV, Coca M, Raymundo RM, Nelson RJ 2001 Genetic structure of Peruvian populations of *Phytophthora infestans*. Phytopathology 91:956–965. 18944122 10.1094/PHYTO.2001.91.10.956

[msv241-B63] Pickrell JK Pritchard JK 2012 Inference of population splits and mixtures from genome-wide allele frequency data. PLoS Genet. 8:e1002967. 23166502 10.1371/journal.pgen.1002967 PMC3499260

[msv241-B64] Pritchard JK Stephens M Donnelly P 2000 Inference of population structure using multilocus genotype data. Genetics 155:945–959. 10835412 10.1093/genetics/155.2.945 PMC1461096

[msv241-B65] Raffaele S Farrer RA, Cano LM, Studholme DJ, MacLean D, Thines M, Jiang RHY, Zody MC, Kunjeti SG, Donofrio NM, 2010 Genome evolution following host jumps in the Irish potato famine pathogen lineage. Science 330:1540–1543. 21148391 10.1126/science.1193070

[msv241-B66] Rieseberg LH Whitton J Linder CR 1996 Molecular marker incongruence in plant hybrid zones and phylogenetic trees. Acta Bot Neerl. 45:243–262.

[msv241-B67] Ristaino JB Groves CT Parra G 2001 PCR amplifications of the Irish potato famine pathogen from historic specimens. Nature 411:695–697. 11395772 10.1038/35079606

[msv241-B68] Rizzo DM Garbelotto M Hansen EM 2005 *Phytophthora ramorum*: integrative research and management of an emerging pathogen in California and Oregon forests. Annu Rev Phytopathol. 43:309–335. 16078887 10.1146/annurev.phyto.42.040803.140418

[msv241-B69] Schierup MH Hein J 2000 Consequences of recombination on traditional phylogenetic analysis. Genetics 156:789–791. 10.1093/genetics/156.2.879 PMC1461297 11014833

[msv241-B70] Serrano-Serrano ML Hernandez-Torres J Castillo-Villamizar G Debouck DG Chacon Sanchez MI 2010 Gene pools in wild Lima bean (*Phaseolus lunatus* L.) from the Americas: evidences for an Andean origin and past migrations. Mol Phylogenet Evol. 54:76–87. 19729069 10.1016/j.ympev.2009.08.028

[msv241-B71] Shimodaira H Hasegawa M 1999 Letter to the Editor: Multiple comparisons of log-likelihoods with applications to phylogenetic inference. Mol Biol Evol. 16:1114–1116.

[msv241-B72] Skotte L Korneliussen TS Albrechtsen A 2013 Estimating individual admixture proportions from next generation sequencing data. Genetics 195:693–702. 24026093 10.1534/genetics.113.154138 PMC3813857

[msv241-B73] Stamatakis S 2006 RAxML-VI-HPC: maximum likelihood-based phylogenetic analyses with thousands of taxa and mixed models. Bioinformatics 22:2688–2690. 16928733 10.1093/bioinformatics/btl446

[msv241-B74] Yang Z 1996 Among-site rate variation and its impact on phylogenetic analyses. Trends Ecol Evol. 11:367–372. 21237881 10.1016/0169-5347(96)10041-0

[msv241-B75] Yoshida K Schuenemann VJ, Cano LM, Pais M, Mishra B, Sharma R, Lanz C, Martin FN, Kamoun S, Krause J, 2013 The rise and fall of the *Phytophthora infestans* lineage that triggered the Irish potato famine. eLife 2:e00731. 23741619 10.7554/eLife.00731 PMC3667578

