# Supplementary material for: Genomic Characterization of a South American Phytophthora Hybrid Mandates Reassessment of the Geographic Origins of Phytophthora infestans
Source: Mol Biol Evol. 2015 Nov 17;33(2):478–91. doi: 10.1093/molbev/msv241 (PMC4866541; doi:10.1093/molbev/msv241)
Supplement: Supplementary Data [file supp_33_2_478__index.html]

Genomic characterization of a South American Phytophthora hybrid mandates reassessment of the geographic origins of Phytophthora infestans — Genomic Characterization of a South American Phytophthora Hybrid Mandates Reassessment of the Geographic Origins of Phytophthora infestans — Genomic Characterization of a South American Phytophthora Hybrid Mandates Reassessment of the Geographic Origins of Phytophthora infestans — Genomic Characterization of a South American Phytophthora Hybrid Mandates Reassessment of the Geographic Origins of Phytophthora infestans — Supplementary Data 

# Genomic Characterization of a South American *Phytophthora* Hybrid Mandates Reassessment of the Geographic Origins of *Phytophthora infestans*

## Supplementary Data

files

- Supplementary Data - zip file
