# Supplementary material for: Genomic Characterization of a South American Phytophthora Hybrid Mandates Reassessment of the Geographic Origins of Phytophthora infestans
Source: Mol Biol Evol. 2015 Nov 17;33(2):478–91. doi: 10.1093/molbev/msv241 (PMC4866541; doi:10.1093/molbev/msv241)
Supplement: Supplementary Data [file supp_msv241_suppl_data.zip › Potato blight genomics SI draft 14.pdf]

# Supplementary Materials and Methods

### *Sample sources and DNA extraction*

The provenance of all samples analyzed in this study is displayed in **table S1**. Although isolate PaX was labeled '*P. infestans* isolate P6096' upon receipt from the World Phytophthora Collection, an isolate with the same identifier was provided to Yoshida *et al*. (2013) for mitogenome analysis. Analysis by Yoshida *et al*. (2013), and our re-analysis of the same sequence data, determined that the mitogenomes of these isolates belong to completely different haplogroups. Throughout our later analyses, we developed further evidence that our sample was mislabeled, as well as strong evidence that it is actually an isolate of *P. andina*. This evidence included its clustering with the three other *P. andina* isolates in the Bayesian analysis of population genetic structuring, the sample's topological position within a clade of the other *P. andina* samples in phylogenetic analysis, and the nearly identical relationship of its phased haplotypes to the other *P. andina* isolates. Based on these criteria, we report this isolate as a *P. andina* of unknown origin (PaX).

### *Illumina blunt-end library preparation and sequencing*

Modern DNA extracts were sheared to a mean fragment length of 200-400bp using a Diagenode Bioruptor XL and then purified using a MinElute PCR purification kit. Sheared DNA extracts were built into Illumina sequencing libraries using a NEBNext DNA Library Preparation for Roche/454 kit and following manufacturers' protocols except that MinElute PCR purification columns were used, rather than a streptavidin beads protocol. Blunt-end adapters P5 and P7 were prepared according to Meyer and Kircher (2010). Initial DNA amplification was carried out through 12 cycles of PCR in 50-μL reactions containing Phusion Hi-Fidelity PCR Master Mix, 0.2 μMol primer inPE1.0, 0.2 μMol Illumina PCR reverse primer with unique index and 5μL library as DNA template. PCR products were purified using MinElute purification columns and size-selected using a Caliper LabChip XT. Libraries were sequenced on an Illumina HiSeq 2000 machine in 150-bp Single Read format for samples P10650, 8143, P6635, P7036, PHU006, 8141, 8844, P6634, P6636, P8144, P6096 and P13803, and in 100-bp Single Read format for samples EC3394, PCO038, PCZ026, PCZ033, PCZ050, PCZ098, PIC97207, PIC97605 and PIC97630. *P. andina* isolate EC3394 was also sequenced on an Illumina GAIIx in SR 76bp format.

Post-extraction and pre-PCR manipulations of historical DNA were carried out in dedicated ancient DNA laboratories at the Centre for GeoGenetics, University of Copenhagen. Historical DNA extracts were built into libraries as described above, except that shearing was not performed and adapter concentration was reduced from manufacturers' specifications during adapter ligation. Unique index sequences were added through PCR amplification of libraries. Initial DNA amplification was carried out through 10 cycles of PCR in two parallel 50-μL reactions containing 5 units AmpliTaq Gold polymerase, 5 μL 1X PCR Gold buffer, 4 mMol $MgCl_2$, 50 μg BSA, 1 mMol each dNTP, 12.5 μMol inPE1.0 forward primer, 10 nMol inPE2.0 reverse primer, 12.5 nMol unique index primer, and 15 μL DNA library template. Amplified PCR products were purified on MinElute purification columns and eluted in 23 μL EB buffer. 5 μL of this purified product was used as template in another round of PCR that was

otherwise the same, except that primer inPE2.0 was not included. These amplified PCR products were purified on MinElute purification columns, size-selected using a Caliper LabChip XT and sequenced on an Illumina HiSeq 2000 machine in 100-bp Single Read format.

### *Residual adapter trimming and read mapping*

Due to the size of our dataset, the command-line pipeline PALEOMIX was used to perform systematic adapter trimming, read mapping, and mapped filtering (Schubert et al. 2014). Low-quality bases and residual adapter sequences (inferred from library building methods or obtained directly from authors) were trimmed from the 3' ends of DNA reads with the software AdapterRemoval v1.5 (Lindgreen 2012) using a mismatch rate of 0.333 (command-line option *--mm 3*). Paired reads overlapping by at least 11bp were collapsed into a single read with a re-calibrated base quality. Reads shorter than 25bp after adapter sequence removal were discarded.

Release ASM14294v1 of the draft reference nuclear genome assembly and annotation of *P. infestans* strain T30-4 (Haas et al. 2009) were obtained from the EnsemblProtists *Phytophthora infestans* assembly and gene annotation database (protists.ensembl.org/Phytophthora_infestans/). Mapping to the reference genome was performed with Burrows-Wheeler Aligner (BWA) v0.5.10 (Li and Durbin 2009) with seeding deactivated and otherwise default settings. PCR duplicates were removed with the MarkDuplicates function within the software Picard v1.82. Collapsed reads were filtered using a script included with PALEOMIX. Reads with mapping quality (MAPQ) scores < 25 were removed from further analysis. Read alignments to the reference genome were optimized with the RealignerTargetCreator and IndelRealigner tools included in the software Genome Analysis Toolkit (GATK) v1.3 (McKenna et al. 2010). Each sample's depth-of-coverage of the reference nuclear genome is shown in **fig. S1**. This pipeline also utilizes MapDamage2 (Jónsson et al. 2013) to recalibrate base qualities of aligned sequence reads in order to remove the residual chemical damage pattern characteristic to ancient DNA. This signal was shown to be quite weak in ancient specimens, however, due to their relatively young age (Martin et al. 2014).

### *Bayesian phylogenetic analysis of mitogenome sequences*

Following Martin *et al.* (2014), reads from all samples were mapped to four mitochondrial reference genomes (Ia, Ib, IIa, IIb; Avila-Adame et al. 2006; Haas et al. 2009), and complete consensus mitogenome sequences for all samples were iteratively assembled from the aligned reads using the software MIA (Green et al. 2008, Burbano et al. 2010). As in Yoshida *et al.* (2013) and Martin *et al.* (2014), a 2.7-kbp region of ambiguous alignment was deleted from the dataset. We used a Bayesian approach to co-estimate the phylogeny and divergence times for a 37.7-kbp alignment comprising 66 *P. infestans* and *P. infestans*-like *P. andina* mitogenome sequences. The mitogenome sequence alignment was partitioned into six subsets: (i) 1st codon sites of the 40 protein-coding genes and open-reading frames; (ii) 2nd codon sites; (iii) 3rd codon sites; (iv) two rRNA genes; (v) 25 tRNA genes; and (vi) intergenic sites. To identify the optimal partitioning scheme for phylogenetic analysis, we ran an exhaustive search in PartitionFinder v1.1.1 (Lanfear et al. 2012). Based on the Bayesian information criterion, we

chose a partitioning scheme that grouped the rRNA genes and tRNA genes together. The ages of the three undated sequences were estimated in the analysis (Shapiro et al. 2011). Posterior distributions of parameters were estimated using Markov chain Monte Carlo (MCMC) sampling. Samples were drawn every 2,000 MCMC steps over a total of 20,000,000 steps. We discarded the first 10% of samples and checked for sufficient sampling. The MCMC analysis was run in duplicate to check for convergence to the stationary distribution.

***Maximum-likelihood phylogenetic analysis of nuclear genomes***

Two alternative methods of genotype calling and sequence alignment were used to confirm the final phylogenetic topology. Firstly, ANGSD v0.588 (Korneliussen et al. 2013) was used in a conservative manner to determine high-quality genotypes (command-line options: *-doMajorMinor 2 -doCounts 1 -GL 1 -minInd 28 -doMaf 1 -SNP_pval 1e-6 -doGeno 5 -baq 1 -C 50 -minQ 10 -setMinDepth 50 -doPost 2 -postCutoff 0.5 -geno_minDepth 2*), then a RAxML ML tree with 100 replicates of fast-bootstrapping using the GTR-GAMMA substitution model with 4 categories (-f a -m GTRGAMMA -p 12345 -# 100 -x 12345) was reconstructed from an alignment of these genotypes (**fig. S2**).

Secondly, the PALEOMIX phylogeny pipeline was used to independently infer a whole-exome phylogeny from alignments of protein-coding sequences. The three lowest-coverage samples (90128, Pi1845B, Pi1882) were excluded from the analysis. Genotype calls at SNP sites for each sample required read depths of $1 \leq C_{i,x} \leq 10C_x$, where $C_x$ is the mean read depth of the sample and $C_{i,x}$ is the read depth of position $i$ in sample $x$. Following this quality filtering, phylogenetic inference was carried out using a super-matrix of protein-coding genes from the *P. infestans* T30-4 genome annotation (Haas et al. 2009) with two partitions (codon positions one and two combined, and position three as a separate group). 100 bootstrap pseudo-replicate alignments were generated from the super-matrix, and parsimony starting trees were generated using RAxML v7.3.2 for both the original super-matrix and the bootstrap super-matrices. Phylogenetic inference was carried out for each super-matrix using ExaML v1.0.2 (Stamatakis and Aberer 2013) under the GAMMA model of nucleotide substitution, using the starting trees generated as described above (**fig. S3**).

***Assessment of nuclear genomic phasing accuracy***

We performed basic assessment of the results of the BEAGLE population frequency-based phasing of *P. andina* nuclear genomic sequences by comparing these computationally phased haplotype sequences to publicly available Sanger sequences derived from cloned PCR products targeting 15 nuclear loci in *P. andina* isolates (Blair et al. 2012). The only *P. andina* isolate shared in common by our study and that of Blair et al. (2012) was P13803, for which phased haplotype sequences for only one locus (560bp of RAS) were available on NCBI. However, Blair *et al*. (2012) provide at least some phased haplotype sequences from other *P. andina* isolates for the following six nuclear loci: RAS, RAS intron, ARP2/3, PUA domain, P4P5K, and Pelota. Haplotype sequences were well conserved between *P. andina* isolates.

From our *P. andina* P13803 Illumina sequence data, we extracted the computationally phased haplotype sequences for each of these loci, manually

aligned them to the corresponding to the Sanger-sequenced haplotypes provided by Blair et al. (2012), and examined sites where SNP variants had been called in our analysis with ANGSD/BEAGLE. Across these loci, a total of 64 heterozygous SNPs were called in our sequences of *P. andina* isolate P13803.

Phasing error was measured as the number of phase-switch error events between SNPs on a haplotype (Lin et al. 2002). Reading from 5' to 3' end of the BEAGLE-phased haplotype sequence, assuming the first SNP to be correctly in phase and thus determining the proper phased haplotype for comparison, we measured how many times the expected allele was shown reported on the other, incorrect haplotype. In this way, in direct comparison with the haplotype sequences from Blair et al. (2012), we observed only a single phasing error in all the analyzed sequences, indicating that our phasing with BEAGLE has an error rate around 1.6% (**table S4**).

### *Bayesian phylogenetic analyses of nuclear genes*

Nucleotide sequences were masked with Ns if not supported by minimum depth of four reads or a variant quality score of at least 50. Heterozygous genotypes were encoded with standard ambiguity codes. Our analyses with BEAST v1.8.0 used the HKY+G model of nucleotide substitution, a strict molecular clock, and a constant-size coalescent prior for the tree. Posterior distributions of parameters were estimated by Markov chain Monte Carlo sampling. Samples were drawn at intervals of $10^3$ over a total of $10^6$ steps. The first 10% of steps were removed as burn-in. Each MCMC analysis was run in duplicate to check for convergence to the stationary distribution. To summarise the date estimates, we examined the precision of the estimates of the root age, representing the coalescence time of all sampled sequences. Precision was measured as the width of the 95% credibility interval divided by the median. The average precision value was 7.7. In order to remove estimates from data sets that might have contained insufficient information, we filtered the estimates according to their precision. We base our interpretation on the date estimates with a precision value of <2. We also report the date estimate for the coalescence of the sequences involved in the historical European outbreak, which we calculated as a proportion of the root age.

### *Four-population tests of introgression*

The same high-confidence genotypes used for the phylogenetic reconstruction (see above) were used to test for introgression. Sample genomes were grouped into the population clusters defined by the NGSadmix analysis. From Durand *et al.* (2011), the population-wise D-statistic is defined as

$$D(P_1, P_2, P_3, P_4) = \frac{\sum_{i=1}^{n}[(1-p_{i1})p_{i2}p_{i3}(1-p_{i4}) - p_{i1}(1-p_{i2})p_{i3}(1-p_{i4})]}{\sum_{i=1}^{n}[(1-p_{i1})p_{i2}p_{i3}(1-p_{i4}) + p_{i1}(1-p_{i2})p_{i3}(1-p_{i4})]},$$

where $n$ is the number of SNPs genotyped in all four populations $P_1$, $P_2$, $P_3$, $P_4$, and $p_{ij}$ is the observed frequency of SNP $i$ in population $P_j$. Standard error was calculated following Green *et al.* (2010) using a block jackknife with discrete 500-kbp blocks. The $P_4$ (outgroup) population consisted of the *P. mirabilis* and *P. ipomoeae* isolates, and only genome positions fixed for the same allele in both outgroup taxa were considered in the analysis.

***Site frequency spectra (SFS) and population genetic summary statistics***
We used ANGSD to compute posterior probabilities of genotypes for all 55 *P. infestans* and *P. andina* sample BAM files at 8,622,136 SNP sites where at least 50% of the individuals passed filters (command-line options *-doGlf 1 -doMajorMinor 2 -doCounts 1 -GL 1 -minInd 28 -doMaf 1 -SNP_pval 1e-6 -doGeno 32 -baq 1 -C 50 -minQ 10 -setMinDepth 50 -doPost 2 -postCutoff 0.5 -geno_minDepth 2*). We then used methods implemented in ngsTools to perform a principle components analysis, first by using these genotype posterior probabilities to the calculate the expected correlation matrix between individuals using SNP sites with a minimum minor allele frequency of 0.05.

For SFS estimation, we used ANGSD to calculate genotype likelihoods from the BAM files for each population cluster separately at high-quality SNP sites where at least 50% of individuals passed filters (command-line options *-minInd 3 -doSaf 1 -doGlf 2 -doMajorMinor 2 -doCounts 1 -GL 1 -doMaf 1 -doGeno 5 -baq 1 -C 50 -minQ 10 -setMinDepth 50 -doPost 2 -postCutoff 0.5 -geno_minDepth 2*). Then, assuming Hardy-Weinberg equilibrium, the population genotype likelihoods were used to estimate the site allele frequency likelihoods and a genome-wide, global SFS for each population cluster. To estimate analyze more conservative spectra not likely to be influenced by mapping errors within inaccessible regions of the *P. infestans* genome, we separately restricted the SFS analysis to uniquely mappable positions described in the main text. We used the methods-of-moments estimator implemented in ngsTools (Fumagalli et al. 2013) to estimate values for whole genome, pairwise population genetic differentiation ($F_{ST}$) from posterior probabilities of population sample allele frequencies of the populations' overlapping sites. Population global site SFS were used as priors. A more conservative estimate of the SFS was generated using only uniquely mappable regions of the reference genome (**fig. S7**).

***Genetic distance within and between genetic clusters***
MEGA 6 (Tamura et al. 2011) was used to determine the pairwise genetic distances between all population clusters using the Maximum Composite Likelihood model (Tamura et al. 2004). After removing two outgroup sequences and masking sites from the master nuclear genome SNP alignment with less than four reads supporting a called genotype, and a minimum genotype quality score of 30, one allele was sampled randomly for each remaining heterozygous genotype. Genetic distance was calculated as an average of the number of base substitutions per site over all sequence pairs between groups from the remaining 3,580,979 positions in the input data. The rate variation among sites was modeled with a gamma distribution (shape parameter = 1). The differences in the composition bias among sequences were considered in evolutionary comparisons (Tamura and Kumar 2002). All ambiguous positions were removed for each sequence pair.

***Estimates of genome heterozygosity and proportions of fixed heterozygotes***
These analyses considered only sites that passed filters for creation of the master nuclear genome SNP alignment. Only sites with ≥ 10 supporting reads were considered for each sample. Samples with values of genomic mean read depth

less than 1.0 were not analyzed. Custom Python scripts were used to tally the proportion of heterozygous genotypes in all genotypes assessed for each sample.

Using the same set of filtered genotypes, custom Python scripts were also used to compute the proportion of SNPs that were fixed as a single heterozygous genotype within each population cluster, for comparison of asexual and sexual lineages. However, genetic clusters with larger sample sizes are more likely to contain an individual with a rare non-fixed genotype (autapomorphous loss-of-heterozygosity mutation) at a particular genomic SNP position. It follows that a simple computation of this proportion might be biased by the sample size, thus reducing the proportion of completely fixed heterozygous positions in larger populations. Instead, for filtered SNP genomic positions with at least two genotypes assessed in the genetic cluster of interest, two of the assessed genotypes were randomly chosen and compared. If the two randomly chosen genotypes matched and were heterozygous (IUPAC ambiguity codes K, M, R, Y, S, or W), the site was called a fixed heterozygote in the genetic cluster. 100 replicates of the analysis were completed to determine the mean proportion of fixed heterozygous positions within each genetic cluster.

***Estimation of extent of linkage disequilibrium***

Seeking evidence of recent sexual reproduction via genetic recombination in our samples, we used the software PLINK v1.09 (Purcell et al. 2007) to examine the decay of genomic linkage disequilibrium within the *P. infestans* and *P. andina* population clusters from biallelic genotypes called with ANGSD. Linkage disequilibrium was approximated by pairwise correlation coefficients between all SNP pairs within 500 kbp. To limit the computational load, only the 50 largest supercontigs were analyzed (57.2% of genome length). Sites with a minor allele frequency < 0.05 were ignored. Correlation coefficients were binned by inter-SNP distance and then plotted using the R package plotrix (Lemon 2006; **fig. S6**).

***Estimation of loss-of-heterozygosity tracts***

In order to investigate loss-of-heterozygosity (LOH) regions expected to arise within a clonal lineage due to mitotic recombination, we employed the genotype likelihood-based, phased and imputed haplotype sequences produced by BEAGLE (described previously). For each clonal lineage, all clonal samples with mean genomic read depth > 2X were considered a cohort. Custom python scripts were used to scan the largest assembled supercontig in the reference genome (supercontig1) for SNP positions where at least one member of the cohort was homozygous for either allele. Progressing from the 5' end of the supercontig, a genomic position was flagged as the beginning of a LOH tract for a particular sample if that sample was homozygous where more than half of the remaining cohort was heterozygous. The termination of a LOH tract was marked at that sample's next heterozygous SNP.

## Supplementary References


Avila-Adame C, Gómez-Alpizar L, Buell RC, Ristaino JB. 2006. Mitochondrial genome sequencing of the haplotypes of the Irish potato famine pathogen, *Phytophthora infestans*. *Current Genetics* **49**: 39-46.

Burbano HA, *et al*. 2010. Targeted investigation of the Neandertal genome by array-based sequence capture. *Science* **328**: 723–5.

Cooke DEL, *et al.* 2012. Genome analyses of an aggressive and invasive lineage of the Irish potato famine pathogen. *PLoS Pathog.* **8**: e1002940.

Durand EY, Patterson N, Reich D, Slatkin M. 2011. Testing for ancient admixture between closely related species. *Molecular Biology and Evolution* **28**: 1–53.

Fumagalli M, *et al.* 2013. Quantifying population genetic differentiation from next-generation sequencing data. *Genetics* **195**, 979–992.

Green RE, *et al*. 2008. A complete Neandertal mitochondrial genome sequence determined by high-throughput sequencing. *Cell* **134**: 416–26.

Green RE, *et al.* 2010. A draft sequence of the Neandertal genome. *Science* **328**: 710–22.

Haas BJ, *et al*. 2009. Genome sequence and analysis of the Irish potato famine pathogen *Phytophthora infestans*. *Nature* **461**: 393–8.

Jónsson H, Ginolhac A, Schubert M, Johnson PLF, Orlando L. 2013. MapDamage2.0: Fast approximate Bayesian estimates of ancient DNA damage parameters. *Bioinformatics* **29**: 1682–4.

Korneliussen TS, Moltke I, Albrechtsen A, Nielsen R. 2013. Calculation of Tajima's D and other neutrality test statistics from low depth next-generation sequencing data. *BMC Bioinformatics* **14**: 289.

Lemon J. 2006. Plotrix: a package in the red light district of R. *R-News* **6**: 8–12.

Li H, Durbin R. 2009. Fast and accurate short read alignment with Burrows-Wheeler Transform. *Bioinformatics* **25**: 1754–60.

Lanfear R, Calcott B, Ho SYW, Guindon S. 2012. PartitionFinder: Combined selection of partitioning schemes and substitution models for phylogenetic analyses. *Molecular Biology and Evolution* **29**: 1695–1701.

Lin S, Cutler DJ, Zwick ME, Chavkravarti A. 2002. Haplotype inference in random population samples. A*merican Journal of Human Genetics* **71**: 1129–37.

Lindgreen S. 2012. AdapterRemoval: easy cleaning of next-generation sequencing reads. *BMC Research Notes* **5**: 337.

Martin MD, *et al*. 2013. Reconstructing genome evolution in historic samples of the Irish potato famine pathogen. *Nature Communications* **4**: doi: 10.1038/ncomms3172.

Martin MD, Ho SYW, Wales N, Ristaino JB, Gilbert MTP. 2014. Persistence of the mitochondrial lineage responsible for the Irish potato famine in extant New World *Phytophthora infestans*. *Molecular Biology and Evolution* **31**: 1414–20.

McKenna A, Hanna M, Banks E, Sivachenko A, Cibulskis K, Kernytsky A, Garimella K, Altshuler D, Gabriel S, Daly M, DePristo MA. 2010. The Genome Analysis Toolkit: a MapReduce framework for analyzing next-generation DNA sequencing data. *Genome Research* **20**: 1297–1303.

Meyer M, Kircher M. 2010. Illumina sequencing library preparation for highly multiplexed target capture and sequencing. *Cold Spring Harbor Protocols 2010*: pdb.prot5448.

Purcell S, Neale B, Todd-Brown K, Thomas L, Ferreira MAR, Bender D, Maller J, Sklar P, de Bakker PIW, Daly MJ, Sham PC. 2007. PLINK: a tool set for whole-genome association and population-based linkage analyses. *American Journal of Human Genetics* **81**: 559–75.

Raffaele S, *et al*. 2010. Genome evolution following host jumps in the Irish potato famine pathogen lineage. *Science* **330**: 1540–3.

Schubert M, *et al.* 2014. Characterization of ancient and modern genomes by SNP detection and phylogenomic and metagenomic analysis using PALEOMIX. *Nature Protocols* **9**: 1056–82.

Shapiro B, Ho SYW, Drummond AJ, Suchard MA, Pybus OG, Rambaut A. 2011. A Bayesian phylogenetic method to estimate unknown sequence ages. *Molecular Biology and Evolution* **28**: 879–87.

Stamatakis A, Aberer AJ. 2013. Novel parallelization schemes for large-scale likelihood-based phylogenetic inference. *2013 IEEE 27th International Symposium on Parallel and Distributed Processing (IPDPS)* **1195**: 20–4.

Tamura K, Kumar S. 2002. Evolutionary distance estimation under heterogeneous substitution pattern among lineages *Molecular Biology and Evolution* **19**: 1727–36.

Tamura K, Nei M, Kumar S (2004) Prospects for inferring very large phylogenies by using the neighbor-joining method. *PNAS* **101**, 11030–11035.

Tamura K, Peterson D, Peterson N, Stecher G, Nei M, Kumar S. 2011. MEGA5: molecular evolutionary genetics analysis using maximum likelihood, evolutionary distance, and maximum parsimony methods. *Molecular Biology and Evolution* **28**: 2731–9.

Yoshida K, *et al*. 2013. The rise and fall of the *Phytophthora infestans* lineage that triggered the Irish potato famine. *eLife* **2**: e00731.

# Supplementary Figures and Tables

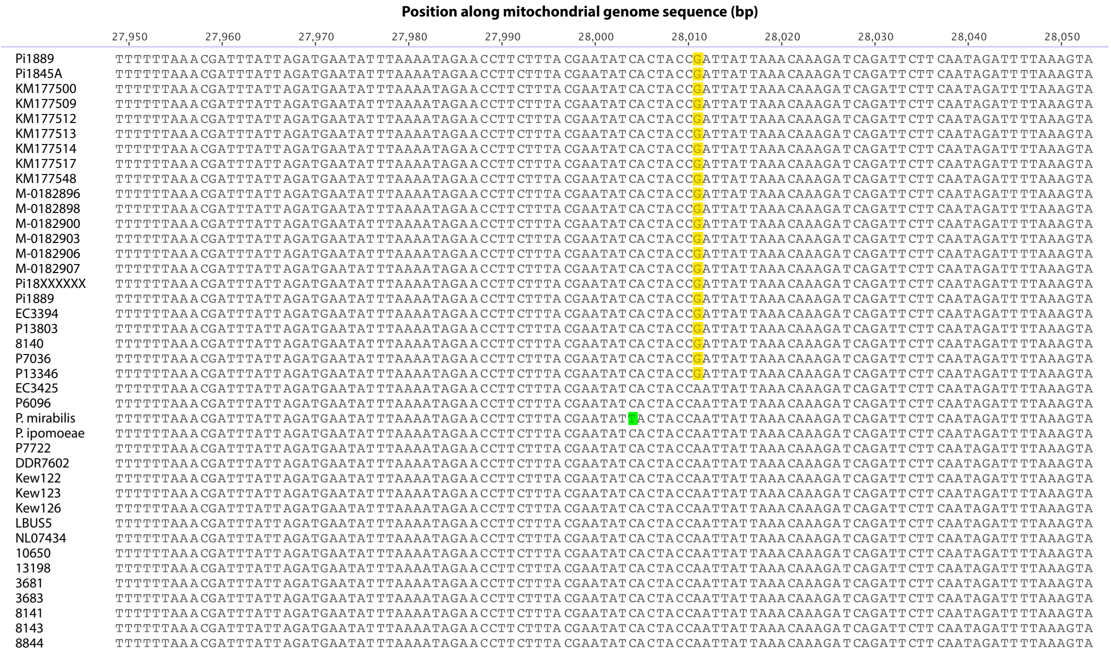


**Fig. S1**. Multiple sequence alignment around a private SNP diagnostic of the HERB-1 mitochondrial lineage. The sample ID for each sequence is provided on the left.

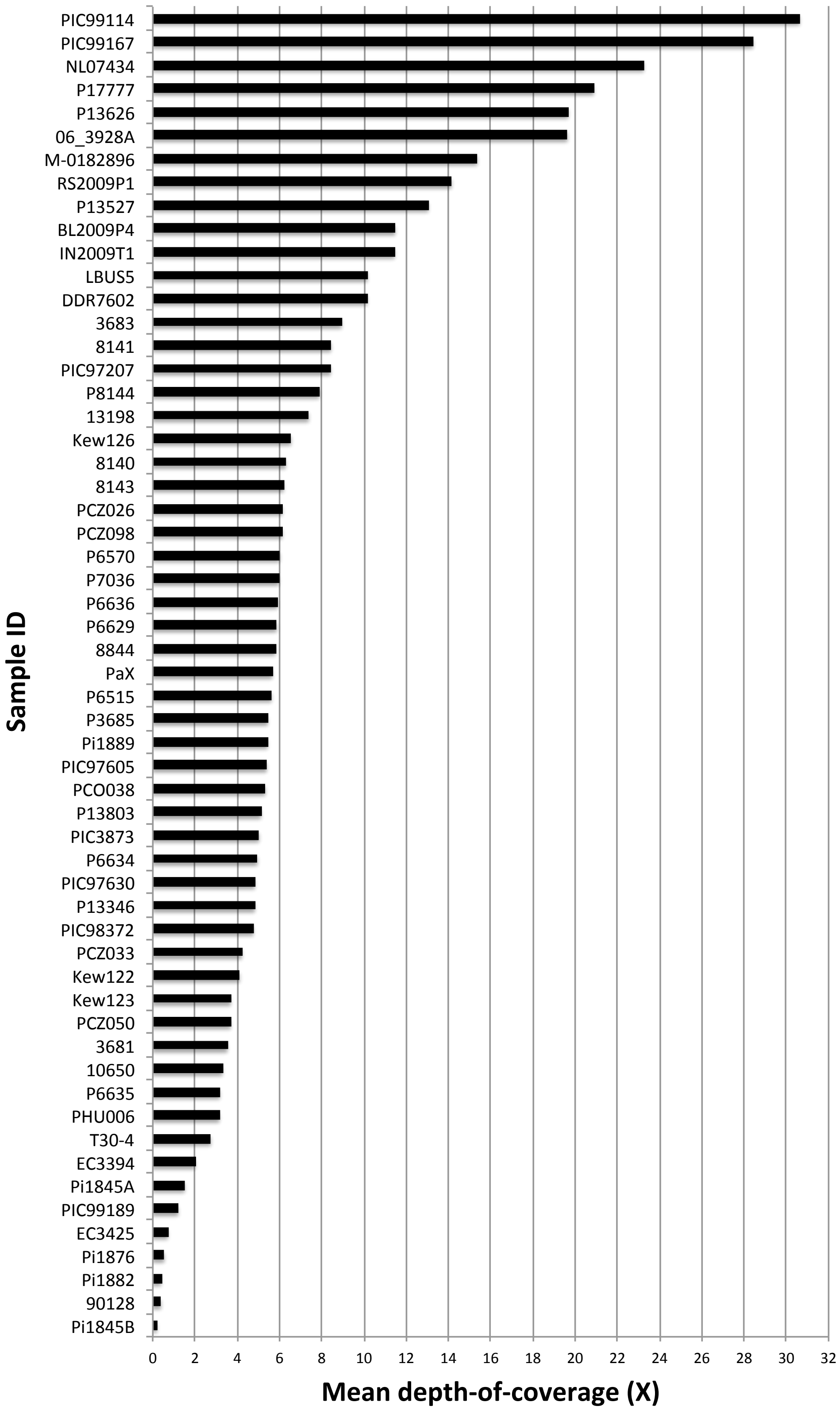


**Fig. S2**. Sample-specific mean depth-of-coverage of the T30-4 reference genome assembly considering only uniquely mapping reads.

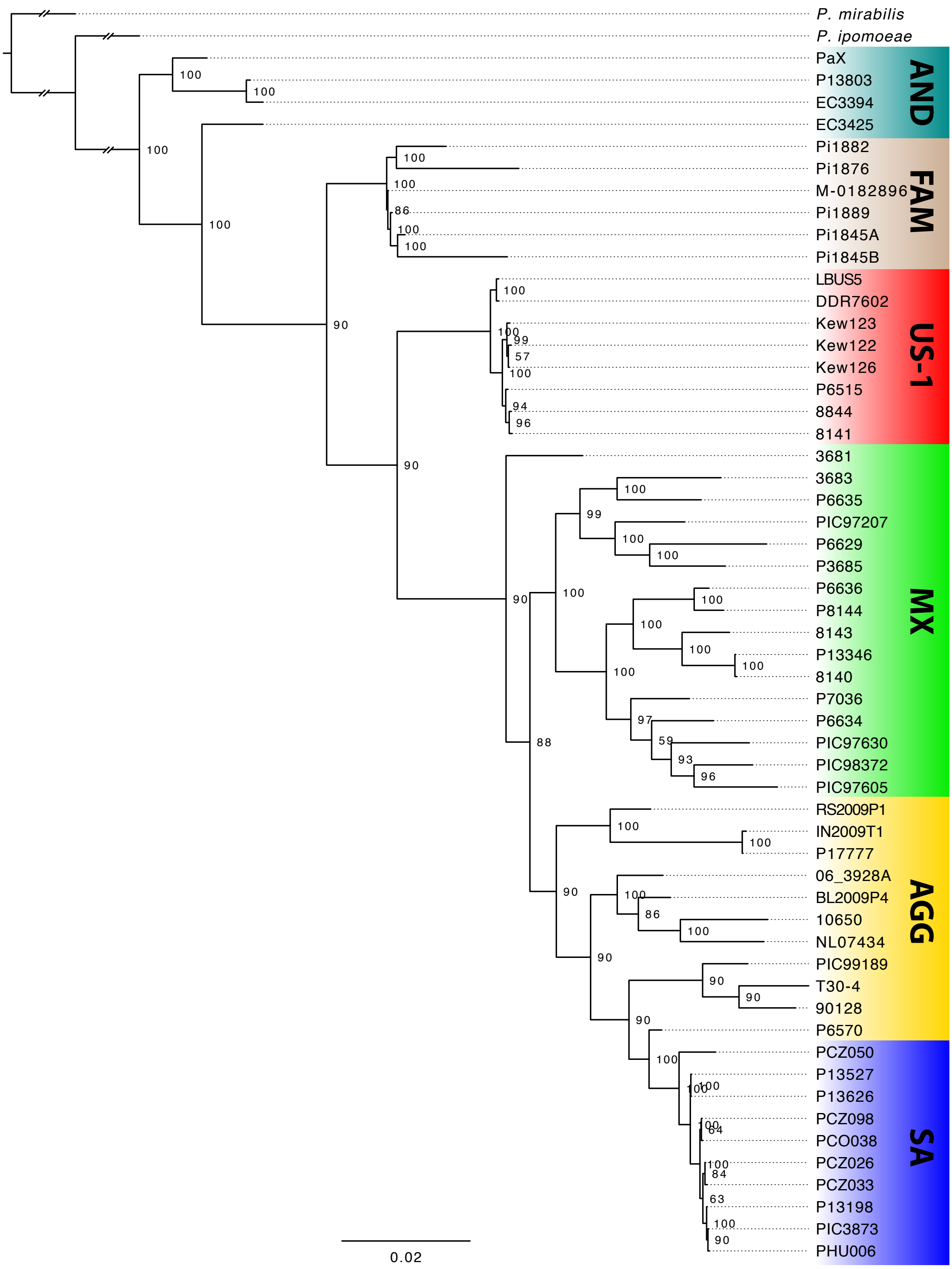


**Fig. S3**. Maximum-likelihood phylogenetic reconstruction of nuclear genome sequences. This is the same phylogenetic reconstruction given in main text fig. 1*c*, except the branch lengths here are untransformed. See Materials and Methods for details on how this tree was estimated. Node support was estimated from 100 bootstrap replicates and is indicated at the nodes. Scale bar is given in nucleotide substitutions per site. Some branches (indicated with two diagonal lines) were truncated in order to better illustrate branch lengths within the samples of *P. infestans* and *P. andina*. The labels for each major clade (AND, FAM, US-1, MX, AGG, SA) are defined in the main text Results, and their shading is consistent with figures in the main text.

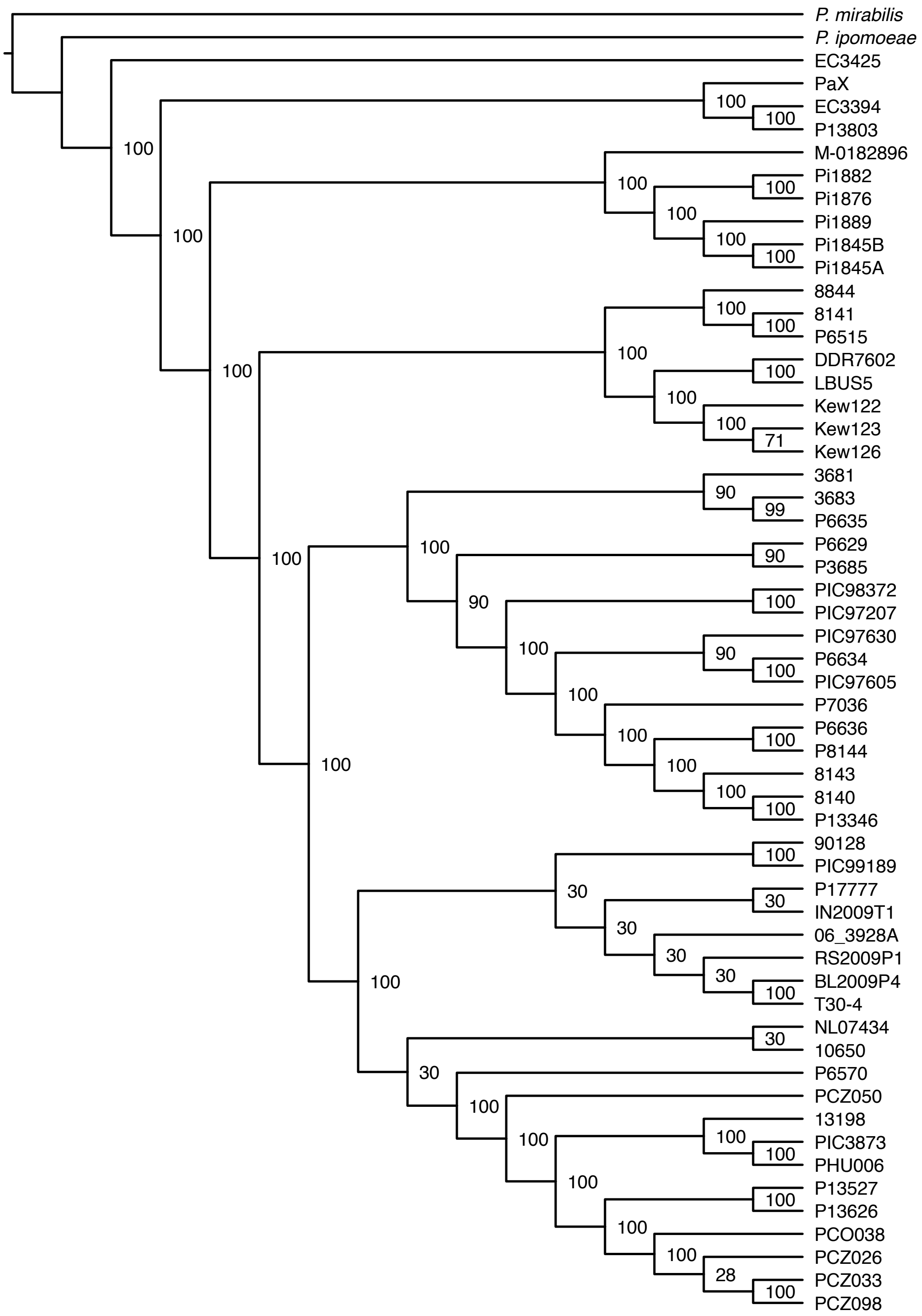


**Fig. S4**. Maximum likelihood phylogeny inferred on genotypes determined with ANGSD. Branch lengths transformed for readability. Bootstrap support values indicated at nodes.

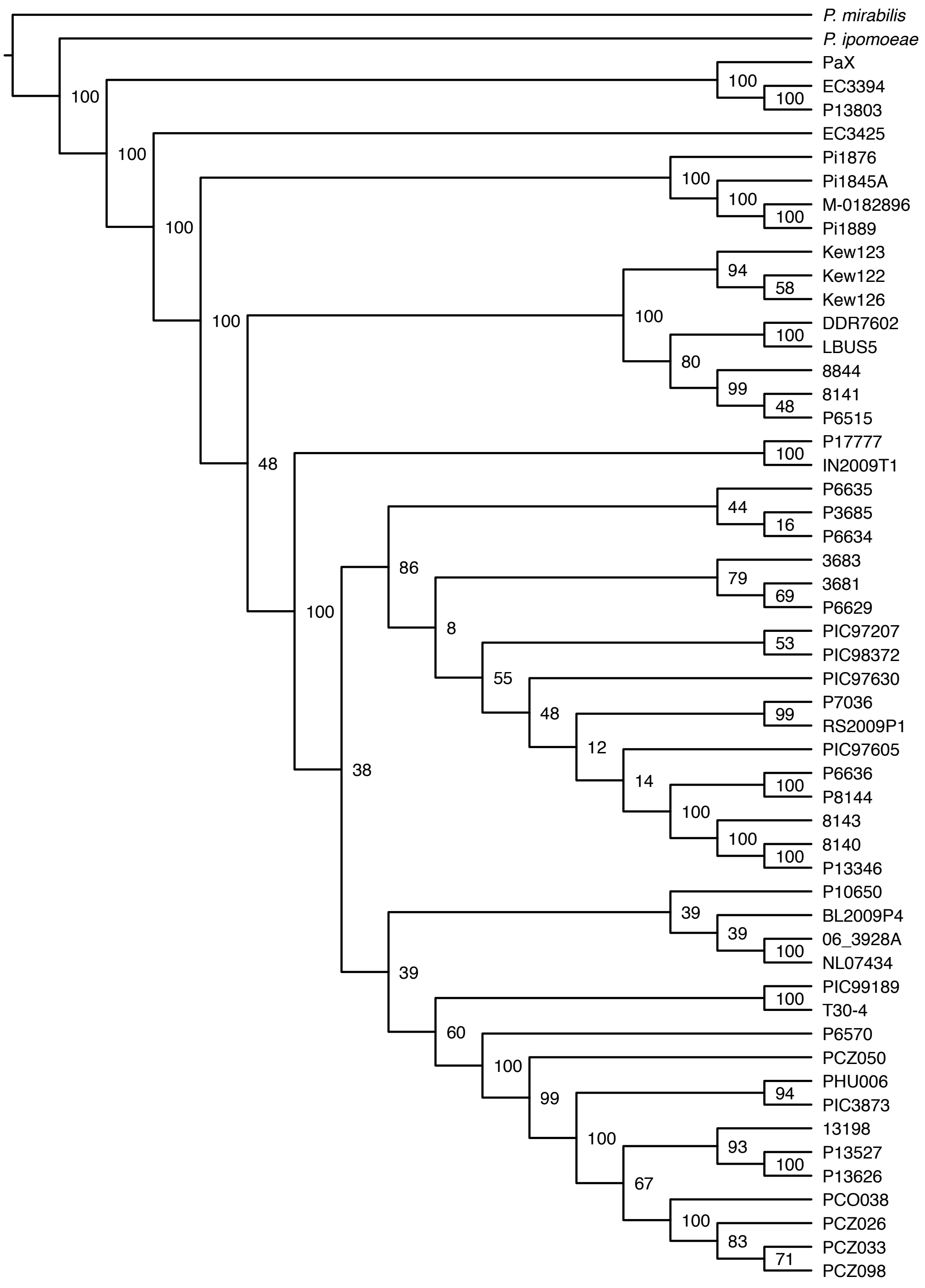


**Fig. S5**. Maximum likelihood phylogeny inferred from alignments of protein coding sequences using PALEOMIX. Branch lengths transformed to increase readability. Bootstrap support values indicated at nodes.

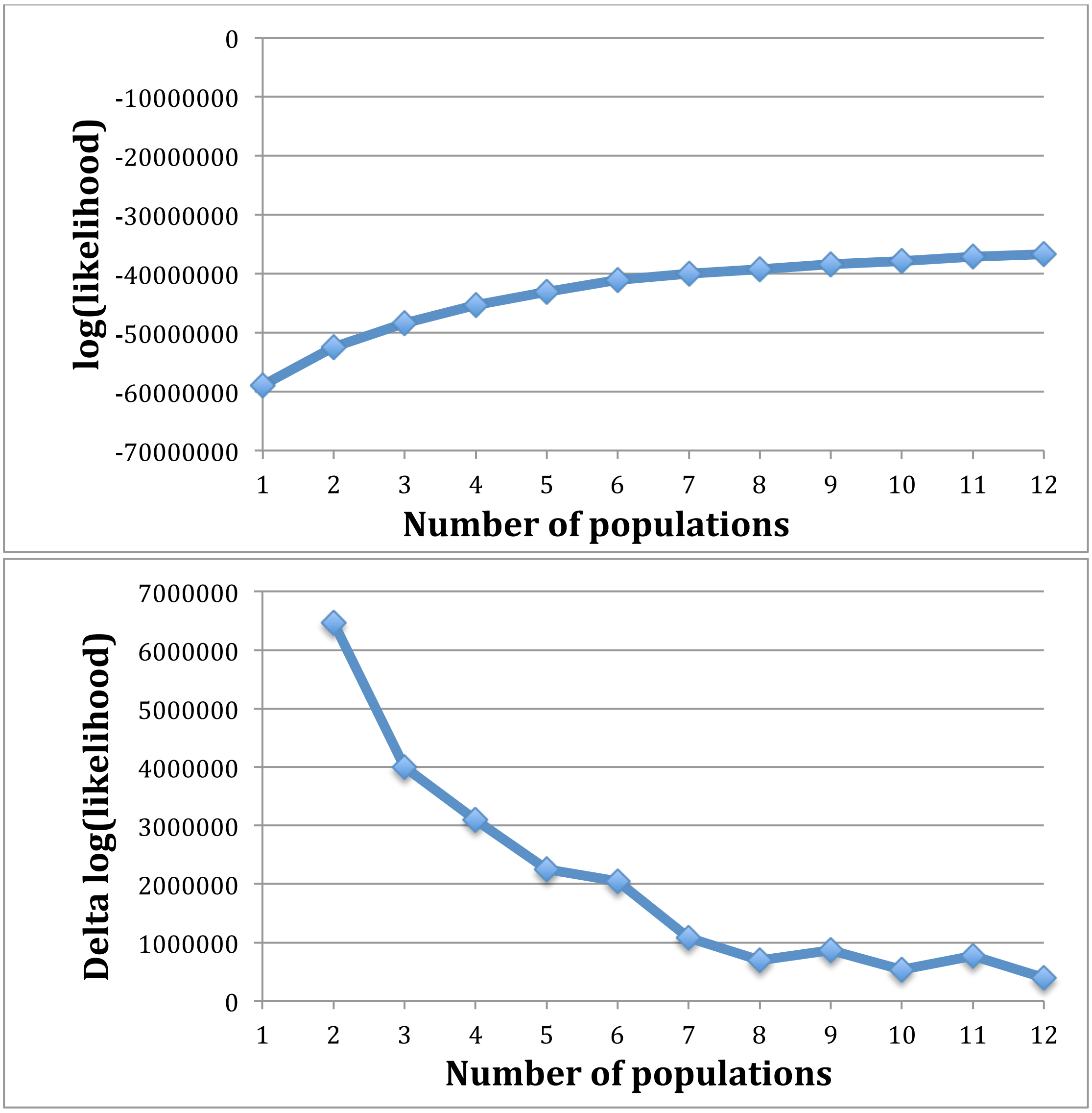


**Fig. S6**. Log-likelihood estimates for values of $K$ = 1 to 12 in the analysis of genetic structure using ngsAdmix. Bottom panel shows change in log-likelihood for increasing $K$.

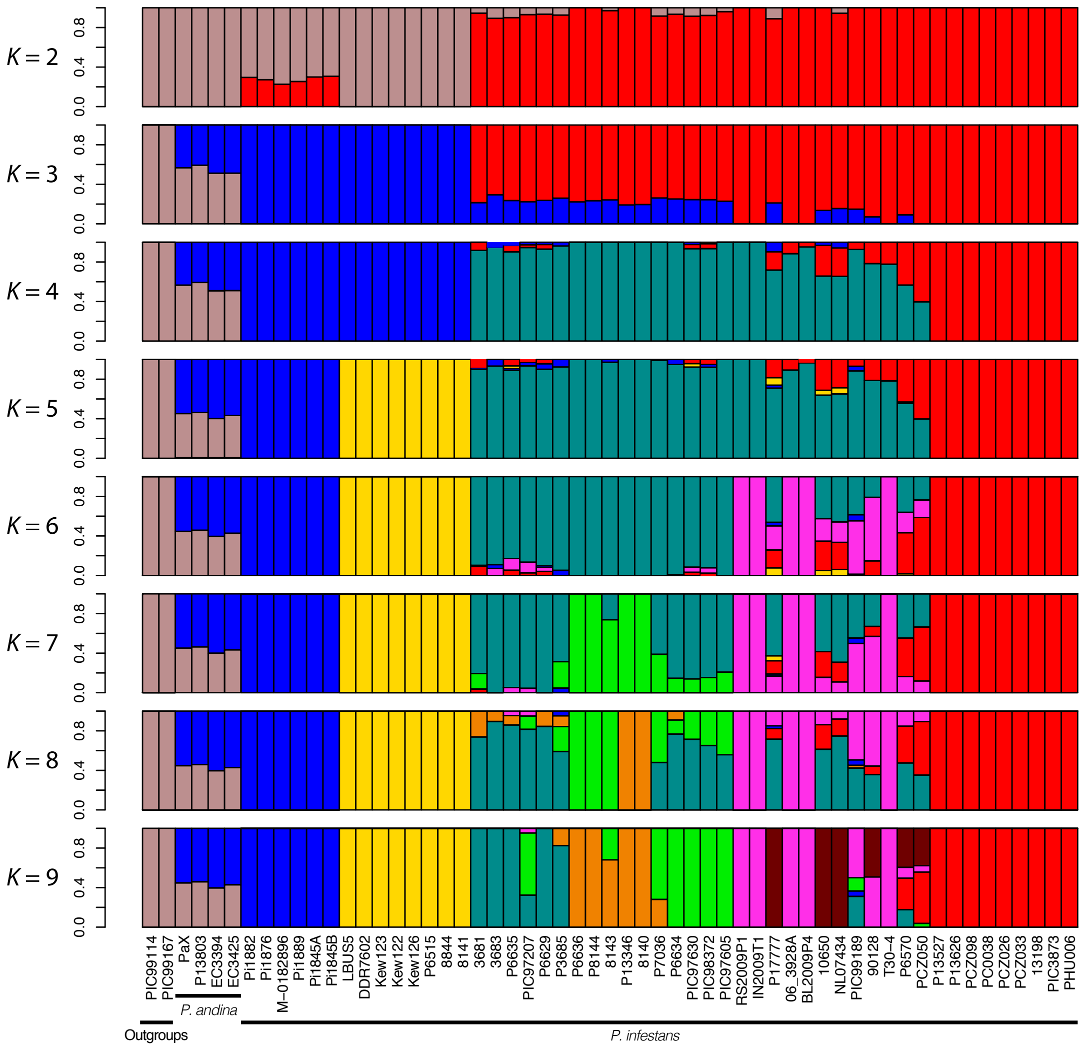


**Fig. S7**. Individual ancestral admixture proportions. Bars indicate the portion of genome ancestry assigned to *K* ancestral populations.

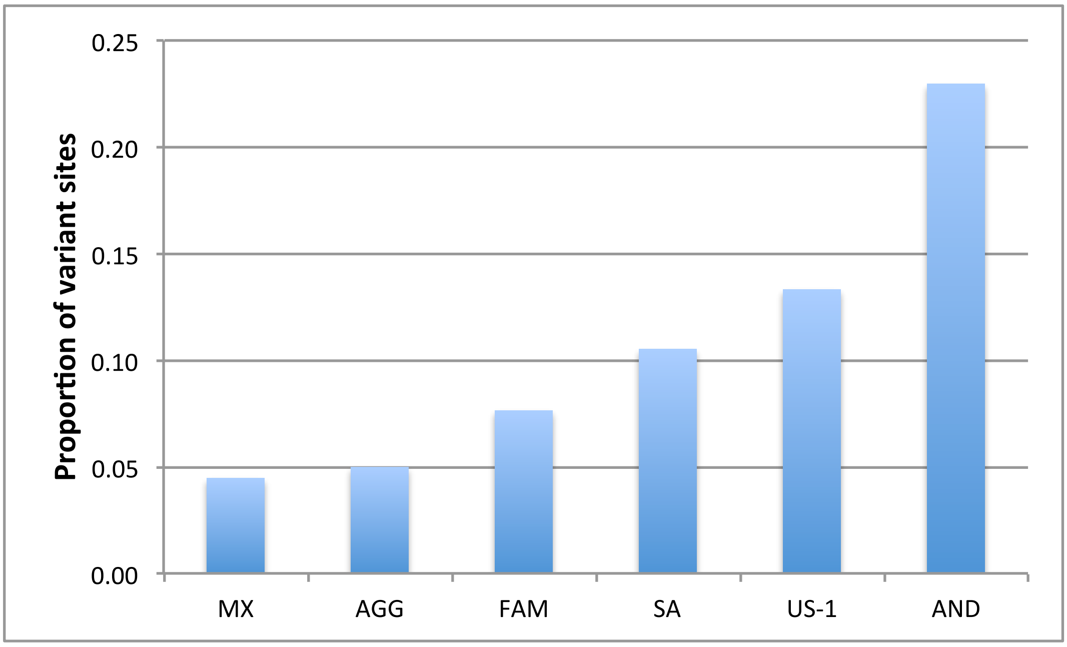


**Fig. S8**. Fraction of variant sites fixed as heterozygotes. Each genetic cluster was considered independently. Labels are consistent with the identification codes assigned to population clusters in fig. 1-5 of the main text.

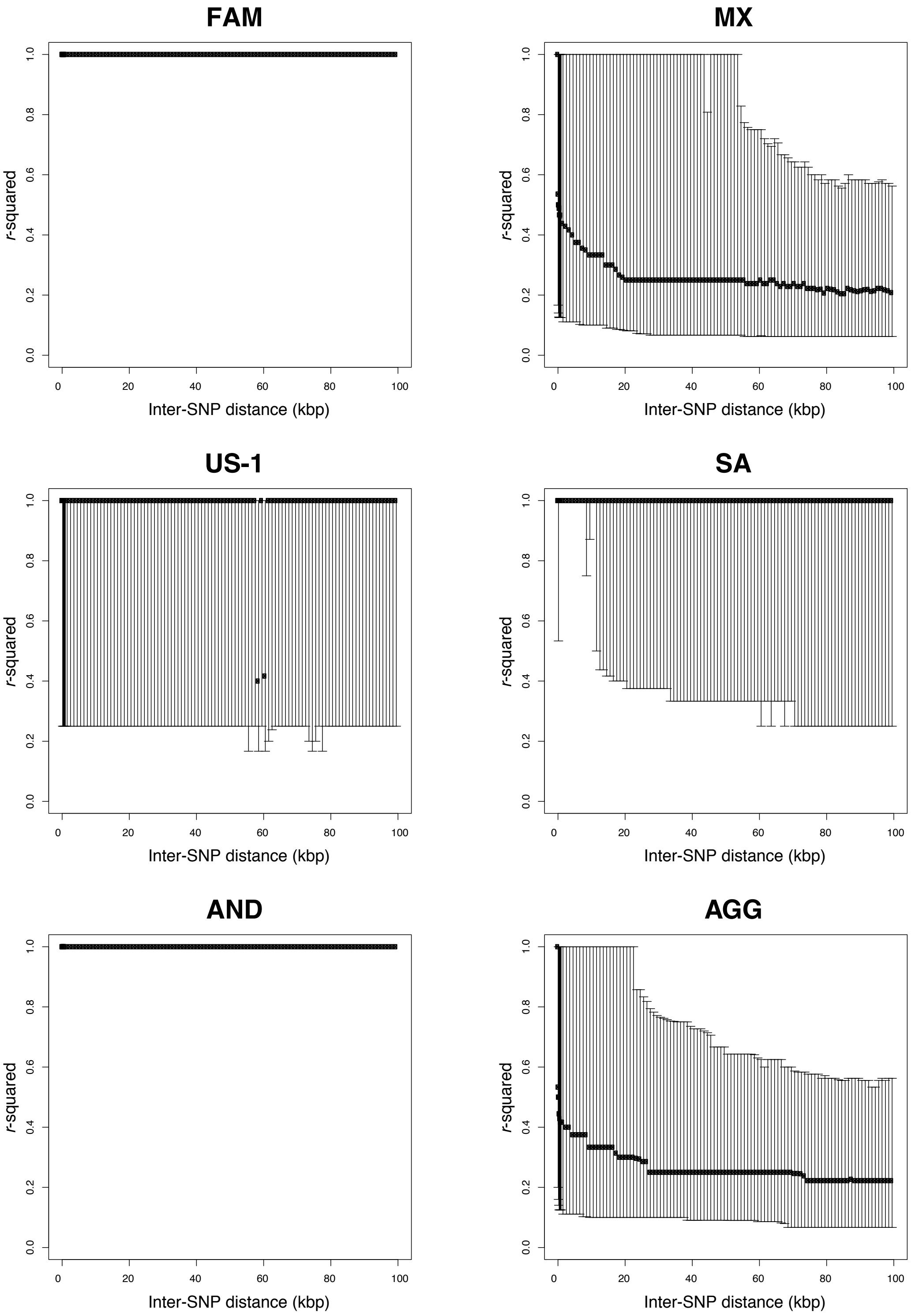


**Fig. S9**. Linkage equilibrium decays rapidly in sexual populations. X-axis, distance (bp). Y-axis, squared-correlation of genotypes. Dots indicate median values for each distance bin, and lower and upper bars show the first and third quartile ranges, respectively.

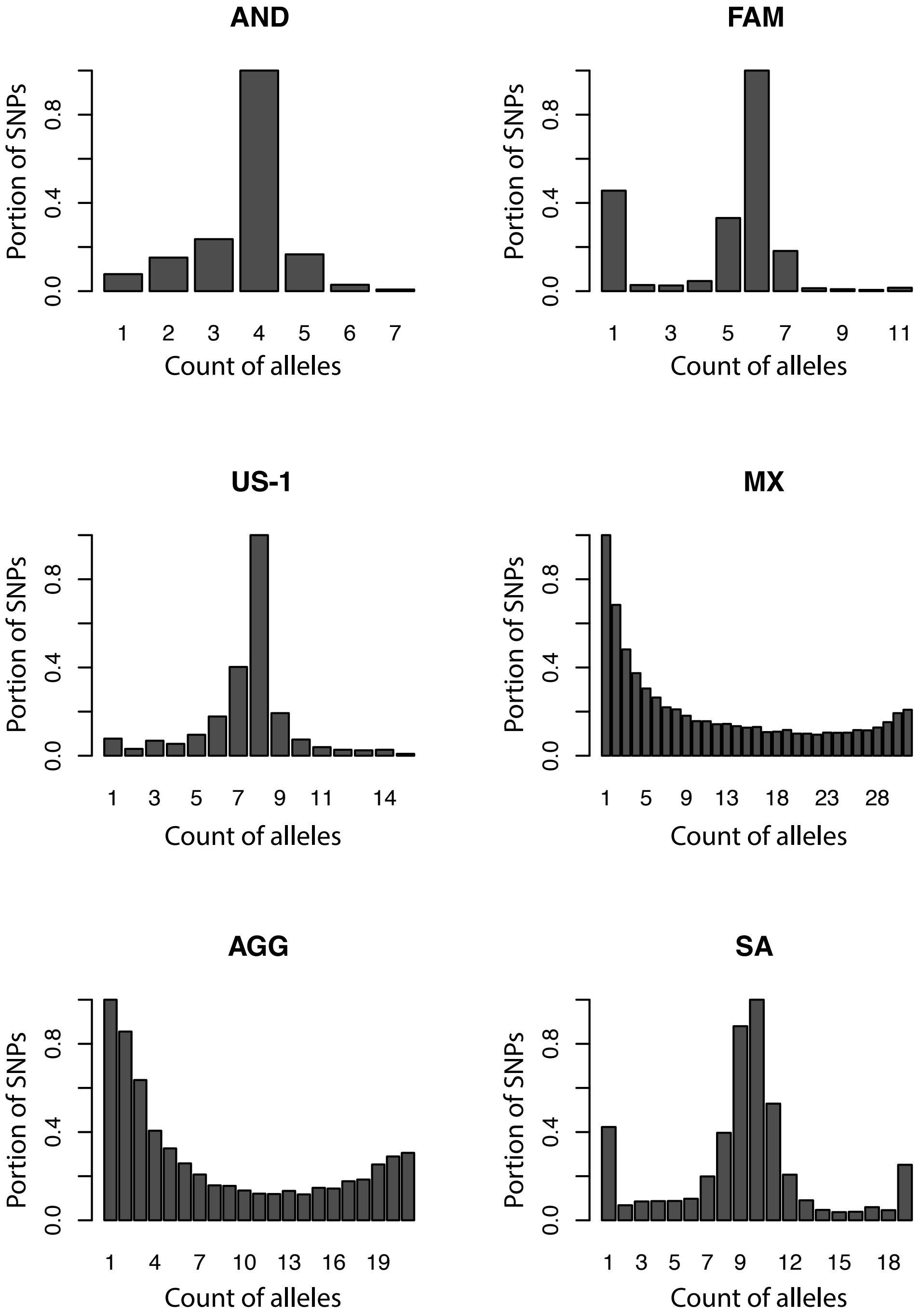


**Fig. S10**. Site frequency spectra for each population cluster considering only in uniquely mappable regions of the reference genome.

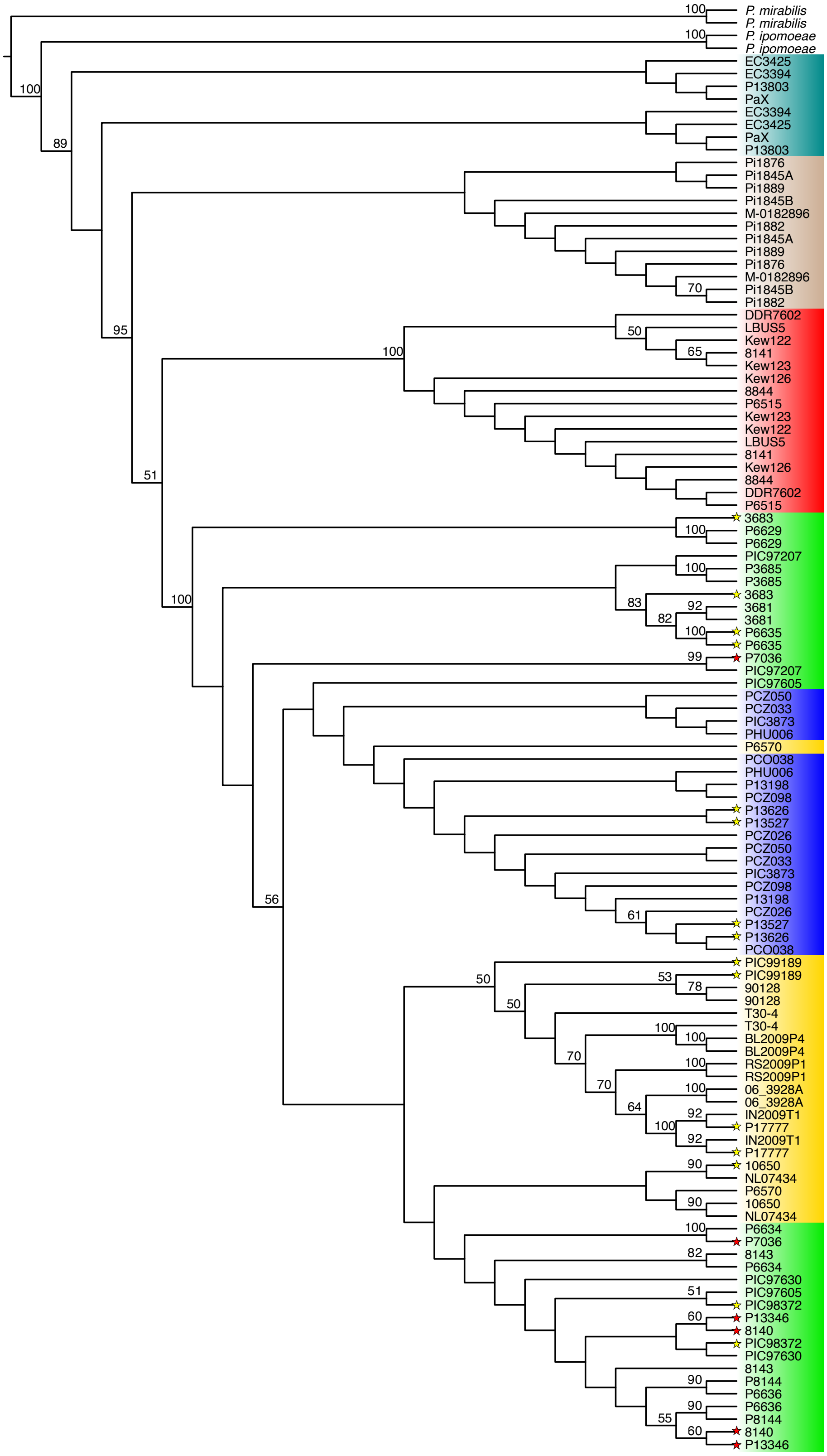


**Fig. S11**. ML tree of phased haplotypes of supercontig2. Branches are transformed for topological clarity. Node labels indicate bootstrap support above 50%. Red stars, HERB-1 mtDNA. Yellow stars, non-*S. tuberosum* host. Colored shading indicates clusters assigned by genetic clustering analysis of the nuclear genome and is consistent with fig. 1 in the main text.

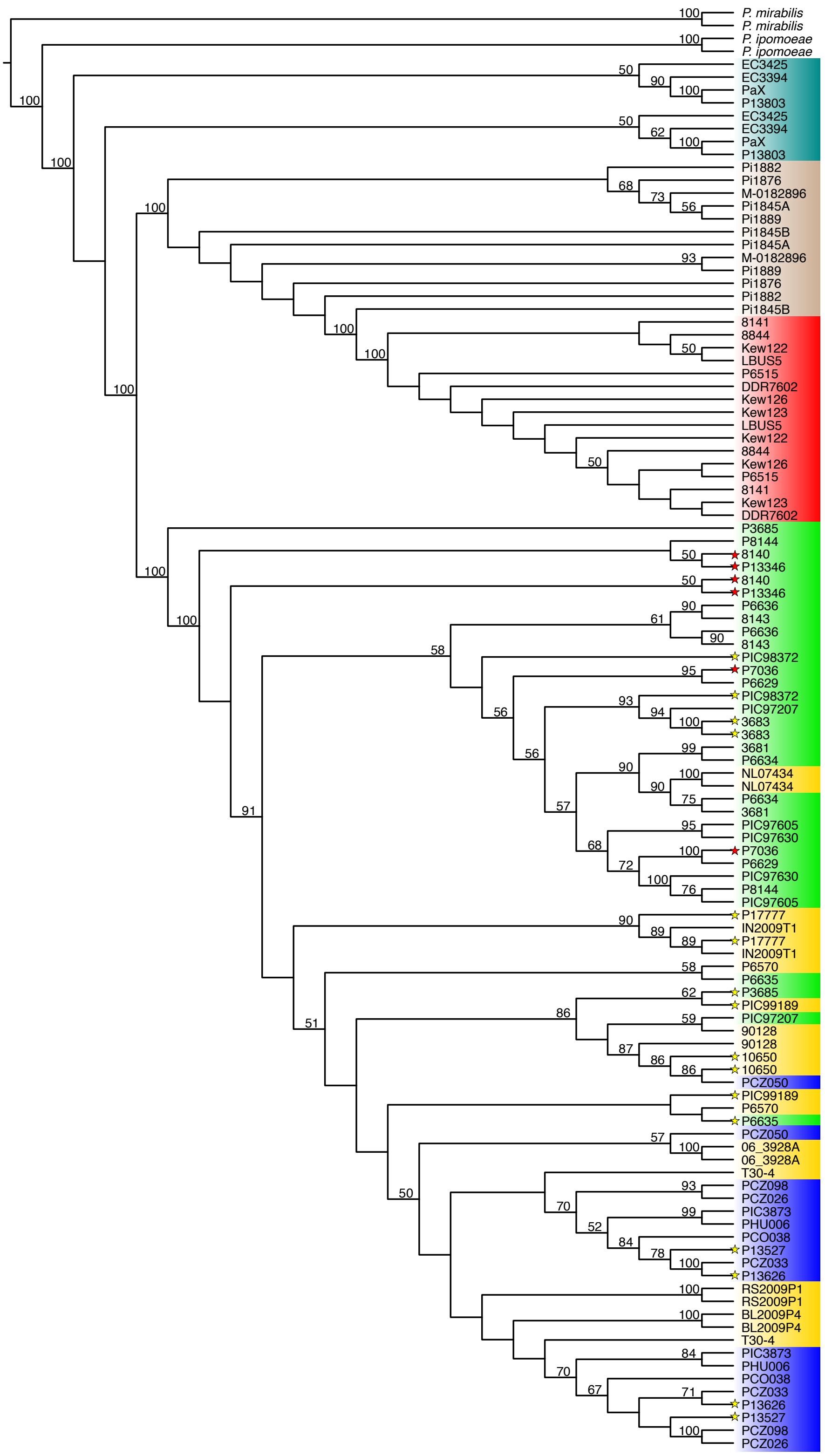

**Fig. S12**. ML tree of phased haplotypes of supercontig3. Branches are transformed for topological clarity. Node labels indicate bootstrap support above 50%. Red stars, HERB-1 mtDNA. Yellow stars, non-*S. tuberosum* host. Colored shading indicates clusters assigned by genetic clustering analysis of the nuclear genome and is consistent with fig. 1 in the main text.

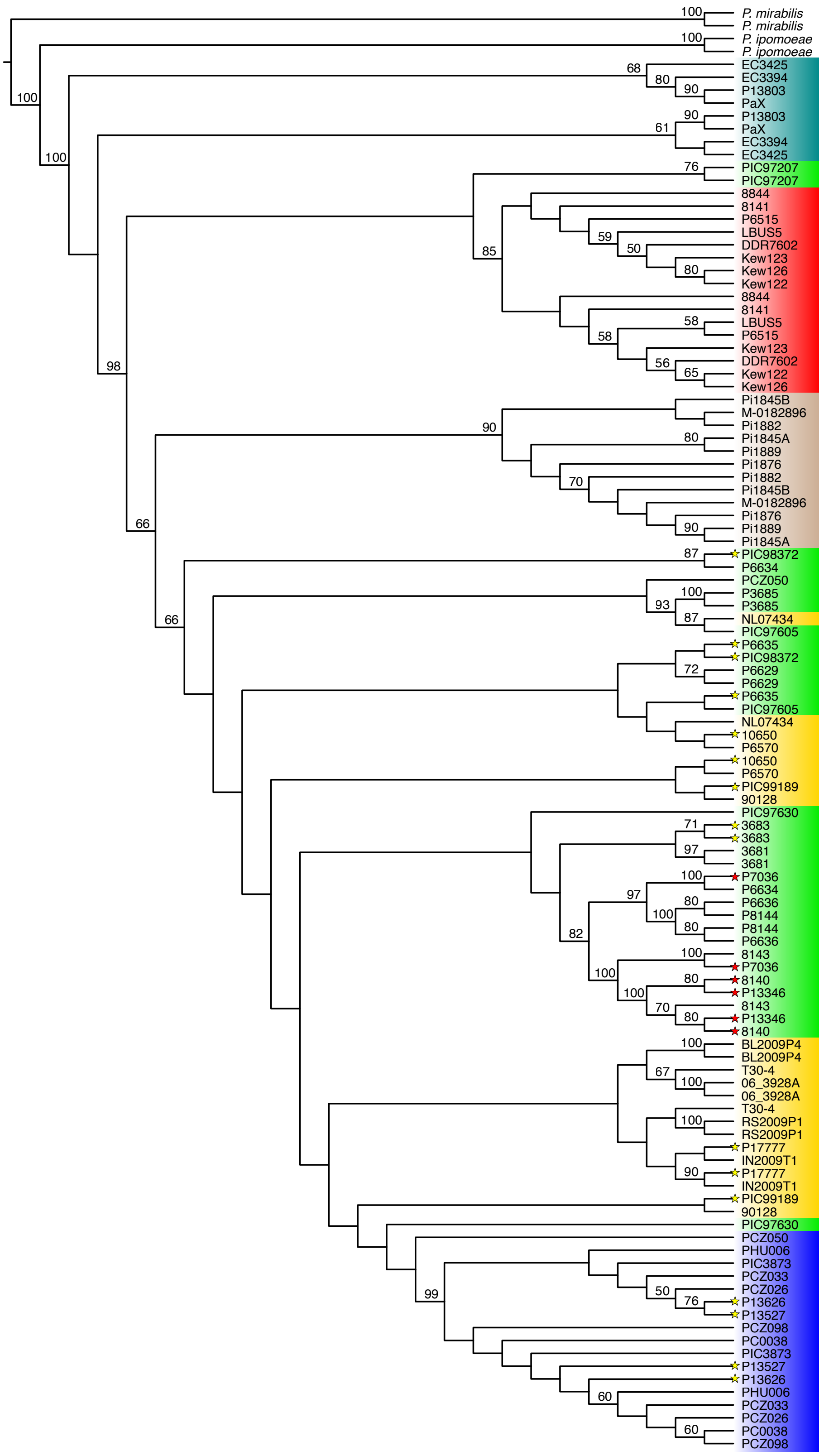


**Fig. S13**. ML tree of phased haplotypes of supercontig4. Branches are transformed for topological clarity. Node labels indicate bootstrap support above 50%. Red stars, HERB-1 mtDNA. Yellow stars, non-*S. tuberosum* host. Colored shading indicates clusters assigned by genetic clustering analysis of the nuclear genome and is consistent with fig. 1 in the main text.

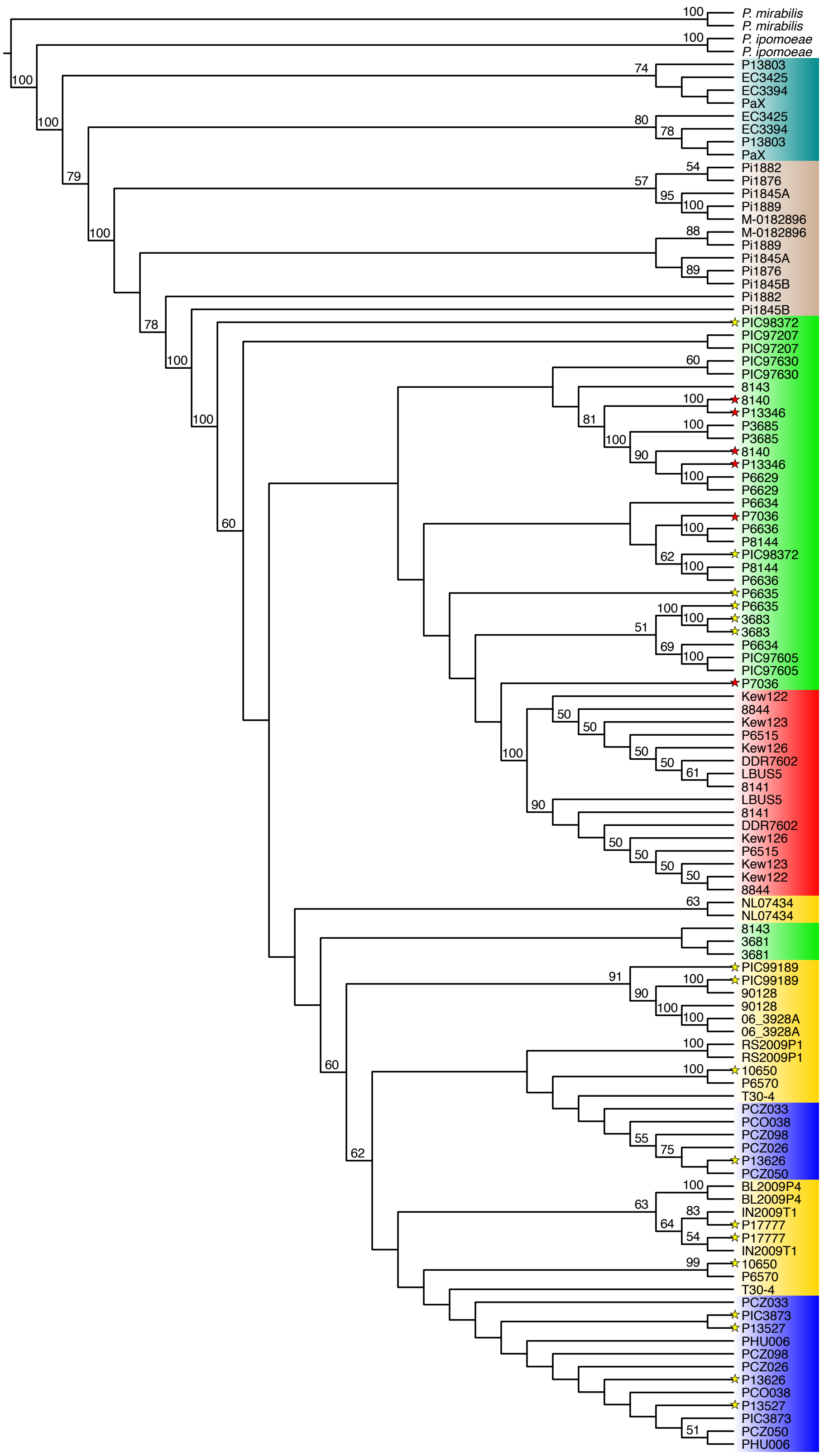


**Fig. S14**. ML tree of phased haplotypes of supercontig5. Branches are transformed for topological clarity. Node labels indicate bootstrap support above 50%. Red stars, HERB-1 mtDNA. Yellow stars, non-*S. tuberosum* host. Colored shading indicates clusters assigned by genetic clustering analysis of the nuclear genome and is consistent with fig. 1 in the main text.

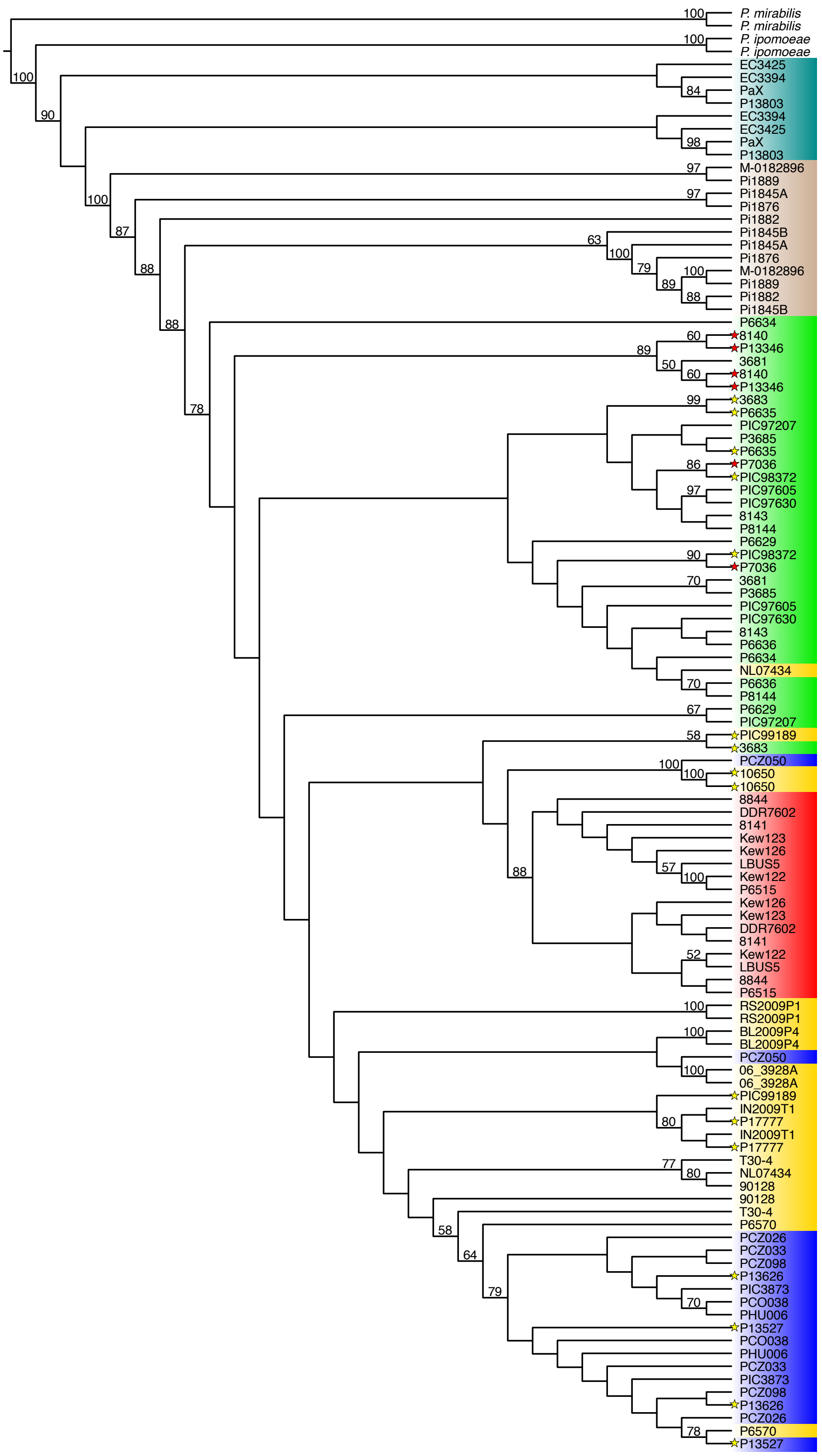


**Fig. S15**. ML tree of phased haplotypes of supercontig6. Branches are transformed for topological clarity. Node labels indicate bootstrap support above 50%. Red stars, HERB-1 mtDNA. Yellow stars, non-*S. tuberosum* host. Colored shading indicates clusters assigned by genetic clustering analysis of the nuclear genome and is consistent with fig. 1 in the main text.

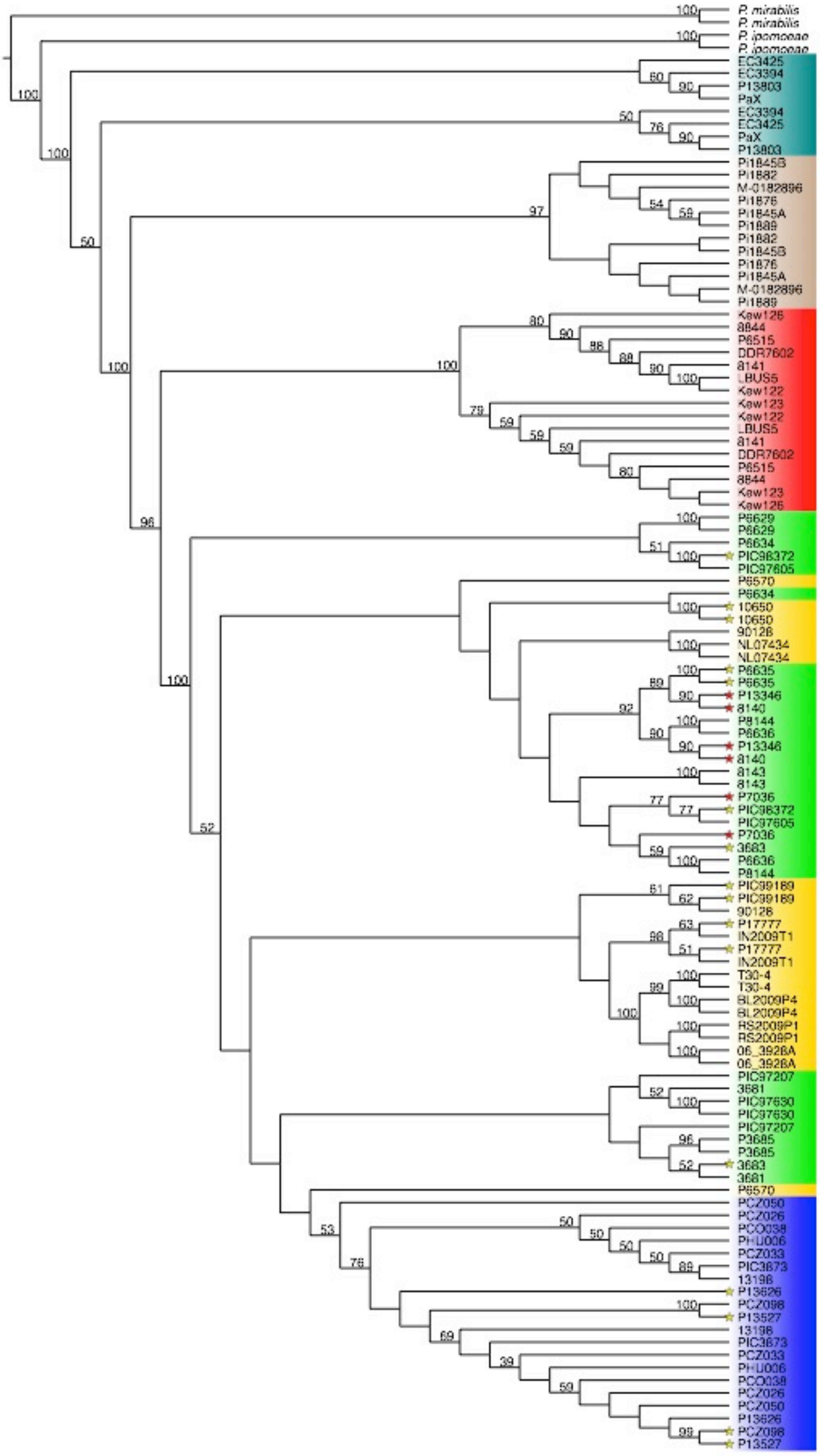


**Fig. S16**. ML tree of phased haplotypes of supercontig7. Branches are transformed for topological clarity. Node labels indicate bootstrap support above 50%. Red stars, HERB-1 mtDNA. Yellow stars, non-*S. tuberosum* host. Colored shading indicates clusters assigned by genetic clustering analysis of the nuclear genome and is consistent with fig. 1 in the main text.

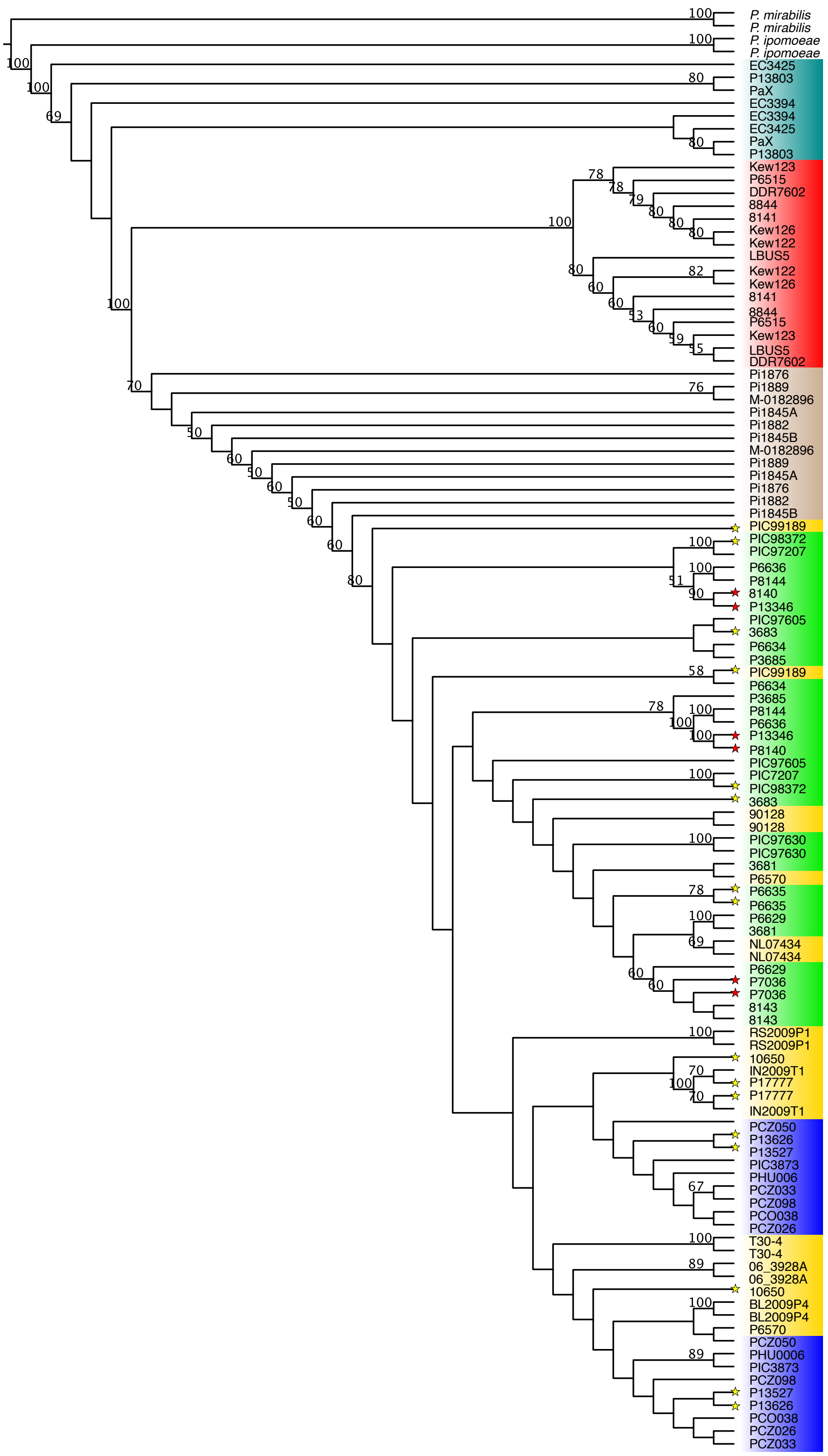


**Fig. S17**. ML tree of phased haplotypes of supercontig8. Branches are transformed for topological clarity. Node labels indicate bootstrap support above 50%. Red stars, HERB-1 mtDNA. Yellow stars, non-*S. tuberosum* host. Colored shading indicates clusters assigned by genetic clustering analysis of the nuclear genome and is consistent with fig. 1 in the main text.

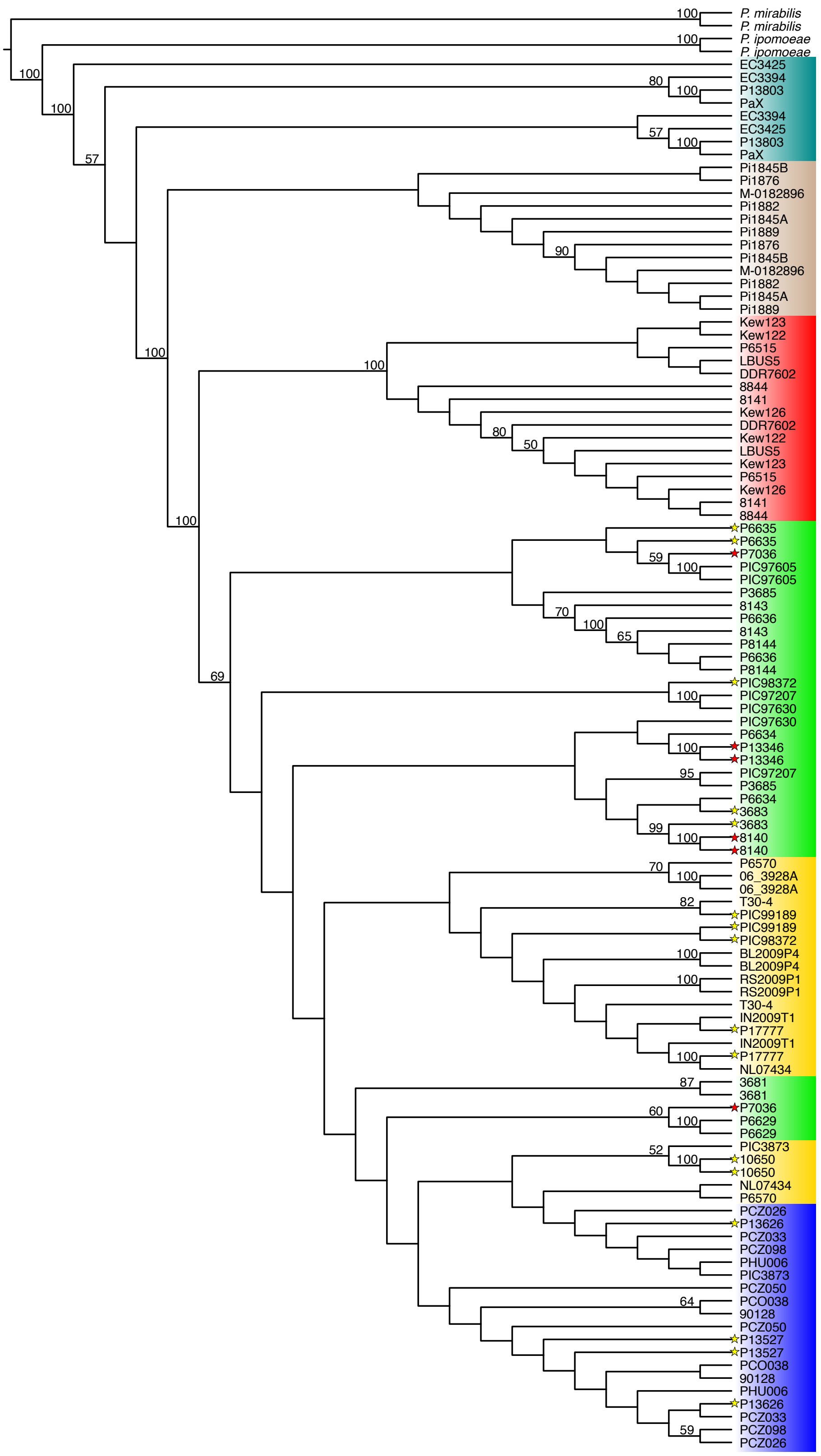


**Fig. S18**. ML tree of phased haplotypes of supercontig9. Branches are transformed for topological clarity. Node labels indicate bootstrap support above 50%. Red stars, HERB-1 mtDNA. Yellow stars, non-*S. tuberosum* host. Colored shading indicates clusters assigned by genetic clustering analysis of the nuclear genome and is consistent with fig. 1 in the main text.

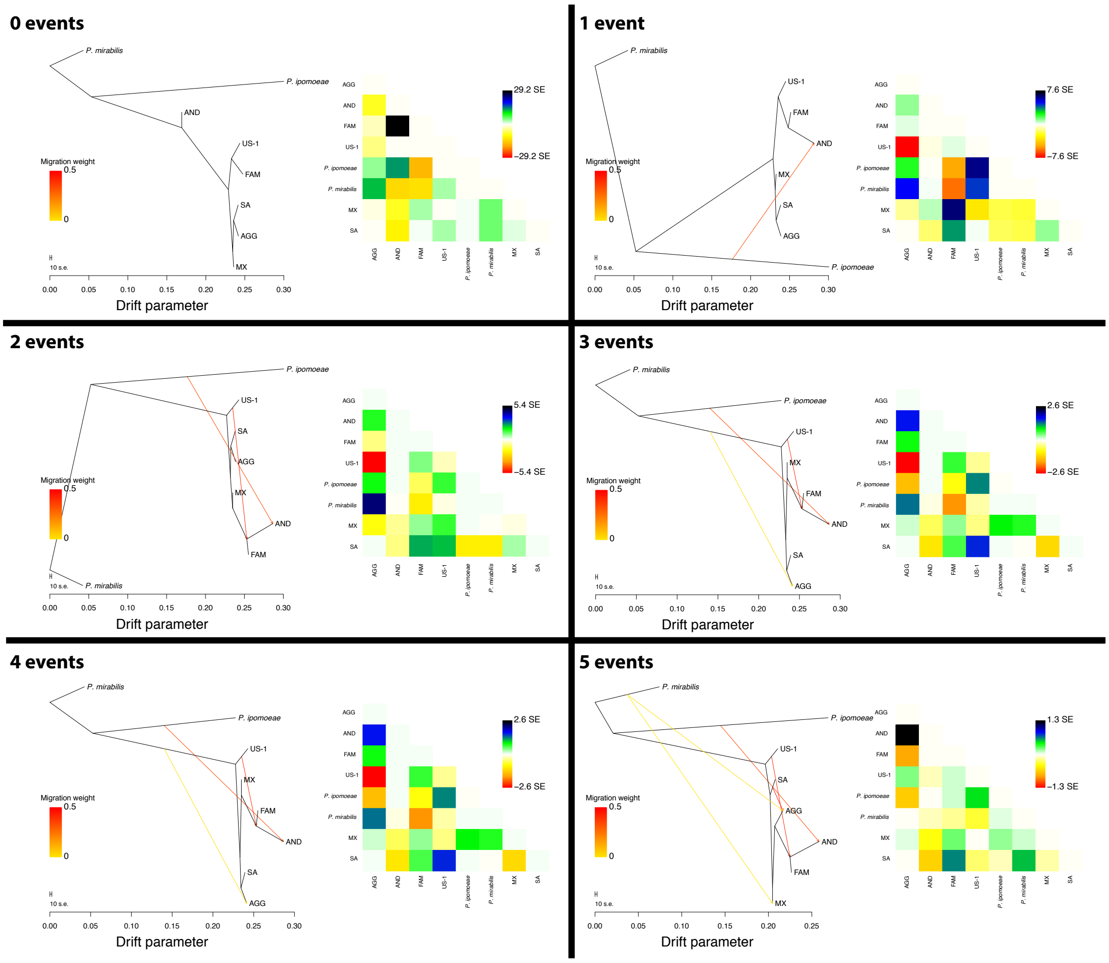

**Fig. S19**. Relationships and migration events between genetic clusters inferred using TreeMix. Each panel shows results for scenarios allowing an increasing number of migration edges (events) from 0 to 5. Each panel shows a maximum-likelihood phylogeny on the left, where the migration weight scale bar represents the fraction of ancestry derived from the migration edge. On the right of each panel is a heat map of the covariance matrix. Large values in this heat map indicate a poor fit of the model to the data. The scale bar represents ten times the average standard error (SE) of the values in the covariance matrix.

**Table S1**. Provenance and information regarding *Phytophthora* spp. samples used in this report. Pi, *Phytophthora infestans*. Pa, *Phytophthora andina*. Pp, *Phytophthora ipomoeae*. Pm, *Phytophthora mirabilis*. ENA, European Nucleotide Archive. M2013, Martin *et al*. (2013). Y2013, Yoshida *et al*. (2013). R2010, Raffaele *et al*. (2010). C2012, Cooke *et al.* (2012). WPC, World *Phytophthora* Collection. H, host herbarium specimen. M, mycelium.

| Sample ID | Alternate IDs | Species | Host | Location | Collection date | Tissue source | RFLP genotype and/or mtDNA lineage | Sequence reads source |
|---|---|---|---|---|---|---|---|---|
| Pi1845A | K91 | Pi | *S. tuberosum* | Audenarde, Belgium | 1845 | H | HERB-1 | M2013 |
| KM177500 | | Pi | *S. tuberosum* | England | 1845 | H | HERB-1 | Y2013 |
| KM177513 | | Pi | *S. tuberosum* | Ireland | 1846 | H | HERB-1 | Y2013 |
| KM177514 | | Pi | *S. tuberosum* | Ireland | 1847 | H | HERB-1 | Y2013 |
| KM177548 | | Pi | *S. tuberosum* | England | 1847 | H | HERB-1 | Y2013 |
| KM177509 | | Pi | *S. tuberosum* | England | 1865 | H | HERB-1 | Y2013 |
| M-0182900 | | Pi | *S. lycopersicum* | Germany | 1873 | H | HERB-1 | Y2013 |
| KM177517 | | Pi | *S. tuberosum* | Wales | 1875 | H | HERB-1 | Y2013 |
| M-0182907 | | Pi | *S. tuberosum* | Germany | 1875 | H | HERB-1 | Y2013 |
| Pi1876 | UPS 1; Up1 | Pi | *S. tuberosum* | Skårop, Denmark | 1876 | H | HERB-1 | M2013 |
| M-0182896 | | Pi | *S. tuberosum* | Germany | 1877 | H | HERB-1 | Y2013 |
| M-0182906 | | Pi | *S. tuberosum* | Germany | 1877 | H | HERB-1 | Y2013 |
| Pi1882 | UPS 2; Up2 | Pi | *S. tuberosum* | Stockholm, Sweden | 1882 | H | HERB-1 | M2013 |
| Pi1889 | K 79 | Pi | *S. tuberosum* | Germany | 1889 | H | HERB-1 | M2013 |
| M-0182903 | | Pi | *S. tuberosum* | Canada | 1896 | H | HERB-1 | Y2013 |
| Kew126 | | Pi | *S. tuberosum* | Britain | 1952 | H | US-1/1b | This report |
| Kew122 | | Pi | *S. tuberosum* | Britain | 1955 | H | US-1/1b | This report |
| Kew123 | | Pi | *S. tuberosum* | Ireland | 1955 | H | US-1/1b | This report |
| M-0182898 | | Pi | *S. tuberosum* | Germany | before 1863 | H | HERB-1 | Y2013 |
| KM177512 | | Pi | *S. tuberosum* | England | Unknown | H | HERB-1 | Y2013 |
| Pi1845B | K16 | Pi | *S. tuberosum* | Great Britain | 1845 | H | HERB-1 | M2013 |
| DDR7602 | | Pi | *S. tuberosum* | Germany | 1976 | M | US-1/1b | Y2013 |
| P1362 | | Pi | *S. tuberosum* | Mexico | 1979 | M | US-1/Ib | Y2013 |
| P8844 | Hohl, Pineda 151 | Pi | *S. tuberosum* | Peru | 1982 | M | US-1/Ib | This report |
| P3681 | Tooley 529 | Pi | *S. tuberosum* | Mexico | 1983 | M | Ia | This report |
| P3683 | Tooley 550 | Pi | *S. stoloniferum* | Mexico | 1983 | M | Ia | This report |
| P3685 | Tooley 533 | Pi | *S. tuberosum* | Mexico | 1983 | M | Ia | This report |
| P6629 | Tooley 511 | Pi | *S. tuberosum* | Mexico | 1983 | M | Ia | This report |
| P6634 | PD_02383; Speil543 | Pi | *S. tuberosum* | Mexico | 1983 | M | Ia | This report |
| P6635 | PD_02388; Fry 580 | Pi | *S. demissum* | Central Mexico | 1986 | M | Ia | This report |
| P7036 | Goodwin 575 | Pi | *S. tuberosum* | Toluca, Mexico | 1986 | M | HERB-1 | This report |
| P8140 | PD_01946; P15103; Fry 572 | Pi | *S. tuberosum* | Toluca, Mexico | 1986 | M | HERB-1 | This report |
| P8141 | Fry 616 | Pi | *S. tuberosum* | Toluca, Mexico | 1987 | M | US-1/1b | This report |

| | | | | | | | | |
|---|---|---|---|---|---|---|---|---|
| P8143 | PD_01949; P15154; Fry 619 | Pi | *S. tuberosum* | Toluca, Mexico | 1987 | M | Ia | This report |
| P8144 | Fry 622 | Pi | *S. tuberosum* | Toluca, Mexico | 1987 | M | Ia | This report |
| T30-4 | | Pi | *S. tuberosum* | Netherlands | 1988 | M | Ia | R2010 |
| P6515 | CIP 27 ; PD_00883; Fry 543 | Pi | *S. tuberosum* | Peru | 1989 | M | US-1/Ib | This report |
| P6570 | Davidse 89018; P570 | Pi | *S. tuberosum* | Netherlands | 1989 | M | IIa | This report |
| 90128 | | Pi | *S. tuberosum* | Netherlands | 1990 | M | Ia | R2010 |
| PHU006 | | Pi | *S. tuberosum* | Peru | 1996 | M | *EC-1*, IIa | This report |
| P12204 | | Pi | *S. tuberosum* | Scotland | 1996 | M | Ia | Y2013 |
| PCO038 | | Pi | *S. tuberosum* | Peru | 1997 | M | *EC-1*, IIa | This report |
| PCZ026 | | Pi | *S. tuberosum* | Cusco, Peru | 1997 | M | *PE-6*, IIa | This report |
| PCZ033 | | Pi | *S. tuberosum* | Cusco, Peru | 1997 | M | *EC1.2*, IIa | This report |
| PCZ050 | | Pi | *S. tuberosum* | Peru | 1997 | M | *PE-3*, Ia | This report |
| PCZ098 | | Pi | *S. tuberosum* | Cusco, Peru | 1997 | M | *EC1.3*, IIa | This report |
| PIC97207 | | Pi | *S. tuberosum* | Mexico | 1997 | M | Ia | This report |
| PIC97605 | | Pi | *S. tuberosum* | Mexico | 1997 | M | Ia | This report |
| PIC97630 | | Pi | *S. tuberosum* | Mexico | 1997 | M | Ia | This report |
| P10650 | MX980099 | Pi | *S. tuberosum* | Toluca, Mexico | 1998 | M | Ia | This report; Y2013 |
| P13198 | CIP3198 tuq | Pi | *S. tuquerrense* | Napo, Ecuador | 1998 | M | *EC-1*, IIa | This report |
| PIC99189 | | Pi | *S. stoloniferum* | Mexico | 1999 | M | Ia | R2010 |
| P13346 | CIP3346 col | Pi | *S. colombianum* | Napo, Ecuador | 2001 | M | HERB-1 | This report |
| P13527 | | Pi | *S. andreanum* | Ecuador | 2002 | M | IIa | Y2013 |
| P10127 | | Pi | *S. lycopersicum* | USA | 2002 | M | IIb | Y2013 |
| P13626 | CIP3626 tbr | Pi | *S. tuberosum* | Ecuador | 2003 | M | IIa | This report |
| LBUS5 | | Pi | *Petunia hybrida* | South Africa | 2005 | M | US-1/Ib | Y2013 |
| P3873 | CIP3873 tbr | Pi | *S. tuberosum* | Cañar, Ecuador | 2005 | M | IIa | This report |
| 06_3928A | | Pi | *S. tuberosum* | England | 2006 | M | *13_A2*, Ia | C2012 |
| NL07434 | | Pi | *S. tuberosum* | Netherlands | 2007 | M | IIa | Y2013 |
| BL2009P4 | PA112 | Pi | *S. tuberosum* | Pennsylvania, USA | 2009 | M | *US-23*, Ia | M2013 |
| IN2009T1 | PA114 | Pi | *S. tuberosum* | Pennsylvania, USA | 2009 | M | *US-22*, Ia | M2013 |
| P17777 | | Pi | *S. lycopersicum* | USA | 2009 | M | *US-22*, Ia | Y2013 |
| RS2009P1 | PA117 | Pi | *S. tuberosum* | Pennsylvania, USA | 2009 | M | *US-8,* Ia | M2013 |
| P6636 | PD_01096; Spelman 618 | Pi | *S. tuberosum* | Toluca, Mexico | 1987 | M | Ia | This report |
| PIC98372 | | Pi | *S. demissum* | Mexico | 1998 | M | Ia | This report |
| P13803 | CIP 3803 | Pa | *S. betaceum* | Pichincha, Ecuador | 2004 | M | *EC-3*, HERB-1 | This report |
| P6096 | CIP 17; Tooley 801 | Pi | *S. tuberosum* | Paurcartambo, Peru | 1984 | M | US-1/Ib | Y2013 |
| PaX | Listed as “P6096” in WPC | Pa | *Solanum* spp. | | | M | Ic | This report |
| EC3394 | EC3394 | Pa | *S. betaceum* | Tungurahua, Ecuador | 2001 | M | *EC-3*, HERB-1 | This report |

| | | | | | | | | |
|---|---|---|---|---|---|---|---|---|
| EC3425 | EC3425 | Pa | *S. brevifolium* | Ecuador | 2001 | M | *EC-2*, Ic | ENA PRJNA52431 |
| PIC99167 | | Pp | *Ipomoeae longipedunculata* | Mexico | | M | | R2010; Y2013 |
| P7722 | | Pm | *S. lycopersicum* | USA | 1992 | M | | Y2013 |
| PIC99114 | | Pm | *Mirabilis jalapa* | Mexico | 1999 | M | | R2010; Y2013 |

**Table S2**. Mean depth of coverage of mitogenome assemblies. (Y) indicates sequence data from Yoshida *et al*. 2013, and (M) indicates sequence data from Martin *et al*. 2013.

| Sample ID | Mean read depth (X) | | Sample ID | Mean read depth (X) |
|---|---|---|---|---|
| 90128 | 78 | | P3681 | 274 |
| 06_3928A | 94 | | P3683 | 237 |
| BL2009P4 | 141 | | P3685 | 278 |
| DDR7602 | 175 | | P6096 | 254 |
| EC3394 | 76 | | P6515 | 272 |
| EC3425 | 12 | | P6570 | 287 |
| IN2009T1 | 140 | | P6629 | 222 |
| Kew122 | 93 | | P6634 | 246 |
| Kew123 | 91 | | P6635 | 226 |
| Kew126 | 70 | | P6636 | 253 |
| KM177500 | 45 | | P7036 | 252 |
| KM177509 | 261 | | P8140 | 294 |
| KM177512 | 176 | | P8141 | 214 |
| KM177513 | 981 | | P8143 | 233 |
| KM177514 | 135 | | P8144 | 261 |
| KM177517 | 240 | | P8844 | 227 |
| KM177548 | 211 | | PaX | 225 |
| LBUS5 | 111 | | PCO038 | 265 |
| M-0182896 | 143 | | PCZ026 | 348 |
| M-0182898 | 206 | | PCZ033 | 260 |
| M-0182900 | 33 | | PCZ050 | 412 |
| M-0182903 | 107 | | PCZ098 | 237 |
| M-0182906 | 142 | | PHU006 | 258 |
| M-0182907 | 164 | | Pi1845A | 58 |
| NL07434 | 88 | | Pi1876 | 137 |
| P10127 | 269 | | Pi1882 | 35 |
| P10650 (M) | 254 | | Pi1889 | 108 |
| P10650 (Y) | 749 | | P3873 | 275 |
| P12204 | 1,013 | | PIC97207 | 1,612 |
| P13198 | 211 | | PIC97605 | 160 |
| P13346 | 273 | | PIC97630 | 165 |
| P13527 | 98 | | PIC98372 | 136 |
| P1362 | 87 | | PIC99189 | 74 |
| P13803 | 226 | | RS2009P1 | 137 |
| P17777 | 127 | | T30-4 | 279 |

**Table S3**. Statistics of loss-of-heterozygosity tracts in clonal lineages. These statistics were calculated along supercontig1 (total length 6,928,287 bp), and measures for the MX and AGG sexual clusters are included for comparison.

| | Sample ID | # tracts | Mean length (bp) | Max length (bp) | Min length (bp) | Total length (bp) | Proportion of analyzed region | Lineage mean proportion |
|---|---|---|---|---|---|---|---|---|
| *US-1* | Kew122 | 120 | 5,251 | 132,581 | 30 | 630,180 | 9.1% | 7.5% |
| | Kew123 | 104 | 7,825 | 165,487 | 2 | 813,845 | 11.7% | |
| | Kew126 | 75 | 5,430 | 38,248 | 2 | 407,302 | 5.9% | |
| | DDR7602 | 79 | 4,116 | 46,183 | 5 | 325,174 | 4.7% | |
| | LBUS5 | 90 | 6,407 | 69,997 | 2 | 576,686 | 8.3% | |
| | P8844 | 71 | 6,023 | 69,997 | 25 | 427,649 | 6.2% | |
| | P8141 | 104 | 4,787 | 38,248 | 2 | 497,933 | 7.2% | |
| | P6515 | 84 | 5,739 | 69,997 | 6 | 482,082 | 7.0% | |
| *SA* | P3873 | 39 | 5,608 | 42,865 | 75 | 218,737 | 3.2% | 6.1% |
| | PCZ026 | 40 | 3,891 | 39,641 | 58 | 155,674 | 2.2% | |
| | PCZ033 | 59 | 8,572 | 88,032 | 2 | 505,762 | 7.3% | |
| | PCZ098 | 53 | 8,356 | 64,853 | 21 | 442,912 | 6.4% | |
| | PC0038 | 61 | 6,164 | 88,032 | 3 | 376,057 | 5.4% | |
| | PCZ050 | 79 | 9,912 | 114,233 | 54 | 783,070 | 11.3% | |
| | P13527 | 72 | 6,826 | 93,192 | 3 | 491,481 | 7.1% | |
| | P13626 | 67 | 6,339 | 93,192 | 12 | 424,739 | 6.1% | |
| | PHU006 | 53 | 10,540 | 88,032 | 3 | 558,649 | 8.1% | |
| | P13198 | 31 | 8,393 | 60,719 | 3 | 260,211 | 3.8% | |
| *AND* | P13803 | 193 | 4,917 | 58,119 | 2 | 949,021 | 13.7% | 14.7% |
| | P6096 | 207 | 5,260 | 81,895 | 2 | 1,088,942 | 15.7% | |
| *FAM* | Pi1889 | 45 | 18,269 | 101,520 | 2 | 822,123 | 11.9% | 9.6% |
| | M-0182896 | 38 | 13,413 | 181,524 | 30 | 509,709 | 7.4% | |
| *MX* | P13346 | 20 | 2,848 | 12,703 | 133 | 56,969 | 0.8% | 1.4% |
| | PIC97207 | 18 | 7,238 | 37,006 | 58 | 130,291 | 1.9% | |
| | PIC97605 | 17 | 4,815 | 28,319 | 10 | 81,859 | 1.2% | |
| | PIC97630 | 25 | 4,557 | 28,319 | 2 | 113,930 | 1.6% | |
| | P6636 | 24 | 5,188 | 17,060 | 125 | 124,528 | 1.8% | |
| | P8143 | 23 | 4,037 | 19,133 | 157 | 92,865 | 1.3% | |
| | P6629 | 22 | 3,851 | 17,060 | 2 | 84,737 | 1.2% | |
| | P6634 | 26 | 2,157 | 10,673 | 163 | 56,105 | 0.8% | |
| | P3681 | 26 | 5,163 | 49,773 | 74 | 134,263 | 1.9% | |
| | P8140 | 15 | 2,975 | 12,703 | 143 | 44,626 | 0.6% | |
| | P6635 | 22 | 4,818 | 37,006 | 2 | 106,012 | 1.5% | |
| | P7036 | 20 | 7,716 | 49,773 | 2 | 154,320 | 2.2% | |
| | P8144 | 23 | 2,105 | 10,673 | 2 | 48,419 | 0.7% | |
| | P3683 | 19 | 4,218 | 22,802 | 299 | 80,160 | 1.2% | |
| | P3685 | 23 | 7,849 | 70,391 | 47 | 180,528 | 2.6% | |
| | PIC98372 | 26 | 2,968 | 17,538 | 74 | 77,168 | 1.1% | |
| *AGG* | IN2009T1 | 9 | 2,082 | 8,757 | 53 | 18,739 | 0.3% | 0.7% |

| | | | | | | | | |
|---|---|---|---|---|---|---|---|---|
| | BL2009P4 | 7 | 8,436 | 38,263 | 1,996 | 59,054 | 0.9% | |
| | RS2009P1 | 6 | 2,022 | 5,198 | 53 | 12,137 | 0.2% | |
| | P90128 | 9 | 4,211 | 12,441 | 75 | 37,901 | 0.5% | |
| | 06_3928A | 9 | 2,646 | 8,757 | 53 | 23,814 | 0.3% | |
| | P17777 | 6 | 7,225 | 26,323 | 2 | 43,352 | 0.6% | |
| | NL07434 | 9 | 9,464 | 20,730 | 348 | 85,178 | 1.2% | |
| | T30-4 | 7 | 3,427 | 10,477 | 2 | 23,993 | 0.3% | |
| | PIC99189 | 9 | 11,524 | 32,294 | 75 | 103,724 | 1.5% | |
| | P10650 | 7 | 11,250 | 32,294 | 1,361 | 78,751 | 1.1% | |
| | P6570 | 9 | 8,510 | 20,730 | 1,001 | 76,598 | 1.1% | |

**Supplementary Table S4**. Details on assessment of computational phasing errors using Sanger sequences.

| Locus ID | NCBI codes for *P. andina* cloned, Sanger sequenced PCR products from Blair et al. (2012) | Length of alignment (bp) | Number of P13803 heterozygous SNPs | Number of haplotype switching errors |
|---|---|---|---|---|
| ARP2/3 | JN673410, JN673411, JN673420, JN673421, JN673416, JN673417, JN673422, JN673423, JN673418, JN673419, JN673426, JN673427, JN673414, JN673415, JN699562, JN699563, JN673424, JN673425 | 985 | 15 | 0 |
| PUA domain | JN673485, JN673486, JN673487, JN673488, JN673491, JN673492, JN673484, JN673489, JN673490, JN673493, JN673494, JN673495 | 645 | 9 | 0 |
| P4P5K | JN678820, JN712902, JN678821, JN678822, JN678823, JN678824 | 1023 | 14 | 0 |
| Pelota | JN678874, JN678875, JN678876, JN678877, JN678878, JN678879, JN712883, JN712884, JN678880, JN678881, JN678882, JN678883, JN678884, JN678885, JN712885, JN712886, JN712887, JN712888 | 744 | 13 | 1 |
| RAS | JN678927, JN678928, JN678929, JN678930, JN678931, JN678934, JN678935, JN678944, JN678945, JN678932, JN678933, JN678942, JN678943, JN678940, JN678941, JN678936, JN678937, JN678938, JN678939 | 559 | 7 | 0 |
| Ras intron | JN679000, JN679001, JN679002, JN679003, JN679008, JN679009, JN679012, JN679013, JN679014, JN679015, JN679010, JN679011, JN679006, JN679007, JN679004, JN679005, JN678998, JN678999 | 308 | 6 | 0 |

**Table S5**. Branch length ratios between nodes A, B and C defined in main text.

| Supercontig | Node A–C distance | Node B–C distance | Factor ($Distance_{A-C}$/$Distance_{B-C}$) |
|---|---|---|---|
| 1 | 0.1737 | 0.0133 | 13.1 |
| 2 | 0.0895 | 0.0107 | 8.4 |
| 3 | 0.1025 | 0.0229 | 4.5 |
| 4 | 0.1232 | 0.0416 | 3.0 |
| 5 | 0.0671 | 0.0316 | 2.1 |
| 6 | 0.0575 | 0.0236 | 2.4 |
| 7 | 0.1387 | 0.0159 | 8.7 |
| 8 | 0.1685 | 0.0461 | 3.7 |
| 9 | 0.1192 | 0.0128 | 9.3 |

**Table S6**. Results of Shimodaira-Hasegawa (SH) testing on bootstrapped phylogenetic analyses of alignments of phased haplotype sequences. We tested the null hypothesis that all tree topologies are equally likely. This table shows the number of trees for which the null hypothesis could not be rejected at the 99% confidence level.

| Supercontig number | Number of equally likely trees |
|---|---|
| 1 | 21 |
| 2 | 14 |
| 3 | 15 |
| 4 | 1 |
| 5 | 5 |
| 6 | 0 |
| 7 | 9 |
| 8 | 0 |
| 9 | 9 |

**Table S7**. Pairwise genetic distances between population clusters. Below diagonal, mean pairwise distances between members of each cluster. Above diagonal, standard error.

| | **MX** | **US-1** | **AND** | **SA** | **FAM** | **AGG** | **Outgroups** |
|---|---|---|---|---|---|---|---|
| **MX** | - | 0.0001 | 0.0003 | 0.0001 | 0.0002 | 0.0001 | 0.0013 |
| **US-1** | 0.0972 | - | 0.0003 | 0.0001 | 0.0002 | 0.0001 | 0.0014 |
| **AND** | 0.1980 | 0.2040 | - | 0.0003 | 0.0004 | 0.0003 | 0.0010 |
| **SA** | 0.0810 | 0.0978 | 0.2037 | - | 0.0002 | 0.0001 | 0.0014 |
| **FAM** | 0.0815 | 0.0842 | 0.1648 | 0.0851 | - | 0.0002 | 0.0022 |
| **AGG** | 0.0785 | 0.0991 | 0.2024 | 0.0753 | 0.0838 | - | 0.0015 |
| **Outgroups** | 1.0119 | 1.0336 | 0.8433 | 1.0269 | 0.9828 | 1.0161 | - |

**Table S8**. Mean genomic $F_{ST}$ for pairwise comparisons of the population clusters considered here.

| | MX | AGG | SA | FAM | US-1 | AND |
|---|---|---|---|---|---|---|
| **MX** | **-** | 0.009 | 0.017 | 0.129 | 0.026 | 0.150 |
| **AGG** | | - | 0.007 | 0.020 | 0.013 | 0.017 |
| **SA** | | | - | 0.076 | 0.050 | 0.078 |
| **FAM** | | | | - | 0.060 | 0.006 |
| **US-1** | | | | | - | 0.074 |
| **AND** | | | | | | - |

**Table S9**. Results of four-population D-tests of introgression. *N*, number of genomic blocks in test. *n*, number of SNPs included in test. *NS*, no significant deviation from zero. *, significant deviation from zero. OUT, outgroup species.

| $P_1$ | $P_2$ | $P_3$ | $P_4$ | Mean D | S.E. | *N* | *n* | Sig? | Interpretation |
|---|---|---|---|---|---|---|---|---|---|
| SA | MX | AND | OUT | 0.0579 | 0.0102 | 2,368 | 1,719,044 | NS | No AND introgression with MX or SA |
| SA | MX | US-1 | OUT | 0.0361 | 0.0096 | 2,648 | 1,568,340 | NS | No US-1 introgression with MX or SA |
| FAM | MX | SA | OUT | 0.0375 | 0.0106 | 2,538 | 1,479,764 | NS | No SA introgression with MX or FAM |
| FAM | SA | MX | OUT | -0.0086 | 0.0106 | 2,558 | 1,479,624 | NS | No MX introgression with FAM or SA |
| FAM | MX | AND | OUT | -0.3560 | 0.0103 | 2,357 | 1,657,196 | * | AND introgression with FAM |
| FAM | SA | AND | OUT | -0.3792 | 0.0106 | 2,278 | 1,454,733 | * | AND introgression with FAM |
| US-1 | MX | AND | OUT | -0.0745 | 0.0098 | 2,374 | 1,745,338 | * | AND introgression with US-1 |
| SA | US-1 | AND | OUT | 0.1171 | 0.0105 | 2,291 | 1,581,504 | * | AND introgression with US-1 |

**Supplementary File 1**. Multiple sequence alignment of iteratively assembled mitogenome sequences in FASTA format. *This file provided as supplementary file upload.*

**Supplementary File 2**. Nuclear genome genes used in the Bayesian dating analysis. 3,676 genes are listed with identifiers that match Haas *et al*. (2009). *This file provided as supplementary file upload.*
